# Inositol phosphates promote HIV-1 assembly and maturation to facilitate viral spread in human CD4^+^ T cells

**DOI:** 10.1371/journal.ppat.1009190

**Published:** 2021-01-21

**Authors:** Gregory A. Sowd, Christopher Aiken

**Affiliations:** Department of Pathology, Microbiology, and Immunology, Vanderbilt University Medical Center, Nashville, United States of America; University of North Carolina at Chapel Hill, UNITED STATES

## Abstract

Gag polymerization with viral RNA at the plasma membrane initiates HIV-1 assembly. Assembly processes are inefficient *in vitro* but are stimulated by inositol (1,3,4,5,6) pentakisphosphate (IP5) and inositol hexakisphosphate (IP6) metabolites. Previous studies have shown that depletion of these inositol phosphate species from HEK293T cells reduced HIV-1 particle production but did not alter the infectivity of the resulting progeny virions. Moreover, HIV-1 substitutions bearing Gag/CA mutations ablating IP6 binding are noninfectious with destabilized viral cores. In this study, we analyzed the effects of cellular depletion of IP5 and IP6 on HIV-1 replication in T cells in which we disrupted the genes encoding the kinases required for IP6 generation, IP5 2-kinase (IPPK) and Inositol Polyphosphate Multikinase (IPMK). Knockout (KO) of IPPK from CEM and MT-4 cells depleted cellular IP6 in both T cell lines, and *IPMK* disruption reduced the levels of both IP5 and IP6. In the KO lines, HIV-1 spread was delayed relative to parental wild-type (WT) cells and was rescued by complementation. Virus release was decreased in all IPPK or IPMK KO lines relative to WT cells. Infected IPMK KO cells exhibited elevated levels of intracellular Gag protein, indicative of impaired particle assembly. IPMK KO compromised virus production to a greater extent than IPPK KO suggesting that IP5 promotes HIV-1 particle assembly in IPPK KO cells. HIV-1 particles released from infected IPPK or IPMK KO cells were less infectious than those from WT cells. These viruses exhibited partially cleaved Gag proteins, decreased virion-associated p24, and higher frequencies of aberrant particles, indicative of a maturation defect. Our data demonstrate that IP6 enhances the quantity and quality of virions produced from T cells, thereby preventing defects in HIV-1 replication.

## Author summary

The discovery that the host cell metabolite inositol hexakisphosphate (IP6) stimulates both immature and mature HIV-1 particle assembly began to uncover the mechanism by which HIV-1 particles become infectious. Inhibitors that block HIV-1 capsid assembly effectively lower HIV-1 production *in cellulo*, however the mechanistic underpinning of how the immature and mature HIV-1 capsids assemble remains enigmatic. We sought to expand upon previous studies and illuminate how IP6, and the related metabolite inositol (1,3,4,5,6) pentakisphosphate (IP5), affect HIV-1 infection. With this goal in mind, we disrupted genes essential for IP5 and/or IP6 production in HIV-1-permissive T cell lines. HIV-1 was less able to propagate in the cells due to defects in intracellular HIV-1 assembly and defects in progeny virion maturation. Our data demonstrate that inositol phosphates are exploited by HIV-1 to orchestrate both virion assembly and maturation. We propose that pharmacological targeting of the HIV-1 inositol phosphate binding pockets in Gag and CA with small molecules will inhibit distinct aspects of HIV-1 replication.

## Introduction

The generation of infectious HIV-1 particles is a complex process that requires assembly of multiple viral proteins, budding from the cellular membrane, and proteolytic maturation. HIV-1 virion assembly is initiated by Gag polymerization at the plasma membrane [1]. Particle assembly requires multiple Gag mediated interactions, including those of the NC domain with viral genomic RNA [2], the MA domain with the plasma membrane [3], and several inter-Gag interactions [4–6]. Upon Gag lattice assembly, the immature virion buds, and Gag is cleaved by the viral protease (PR) [1,7] liberating CA and other viral proteins which reorganize to form a mature conical core containing the functional viral capsid. Perturbations of assembly, release, or maturation are deleterious to HIV-1 infection. Specifically, substitutions in Gag affecting inter-Gag interactions can lower overall infectivity, decrease virion-associated p24, reduce virus release efficiency, disrupt viral maturation, and increase the proportion of immature virus particles [4,6,8–10]. Thus, proper Gag assembly is critical to form infectious HIV-1 virions.

The cellular metabolite inositol hexakisphosphate (IP6) binds both to the SP1 domain of HIV-1 Gag [11] and arginine 18 (R18) of CA [12]. IP6 interacts with Gag in the region of the SP1 six-helix bundle, thereby promoting lattice assembly and stabilizing it [11,13]. Consistently, addition of the related metabolite inositol (1,3,4,5,6) pentakisphosphate (IP5) or IP6 to recombinant Gag stimulates immature particle formation *in vitro* [11,14,15], an effect that has also been observed with BIV, FIV, EIAV, SIV, and HIV-2 Gag proteins [16] implying that this is a general mechanism of assembly employed by lentiviruses.

Upon proteolytic processing of Gag during maturation, the 6-helix bundle is destroyed and, IP6 is thought to subsequently bind to Arg18 at the center of the CA hexamer [12]. IP6 and IP5 can bind to CA hexamers *in vitro* [17] and promote CA mature cone-like particle formation *in vitro* [11]. Moreover, IP6 promotes the conversion of immature virus-like particles assembled from Gag and viral Ψ RNA into mature-like capsid lattices upon cleavage by PR *in vitro* [15]. CA mutants that are impaired in IP6 binding exhibit lower capsid stabilities than wild-type (WT) HIV-1 [17,18], and addition of exogenously added IP6 to HIV-1 cores prevents core disassembly [12]. Addition of IP6 to purified HIV-1 cores protects viral cDNA from degradation by DNaseI during reverse transcription *in vitro*, suggesting that the metabolite might influence reverse transcription in target cells by stabilizing the viral capsid [12]. Moreover, IP6 promotes endogenous reverse transcription within the viral core *in vitro* [19,20]. These observations are consistent with a model wherein IP6 promotes Gag assembly and the subsequent assembly of CA during virus maturation by stabilizing both immature Gag and mature CA lattices [11].

Inositol phosphates, including IP5 and IP6, regulate diverse cellular processes including (but not limited to) transcription [21], calcium signaling [22], and necroptosis [23]. IP5 and IP6 are formed as part of an enzymatic cascade initiated by the cleavage of phosphatidylinositol (4, 5) bisphosphate to form inositol (1, 4, 5) trisphosphate (IP3) [22]. IP3 is phosphorylated by Inositol Polyphosphate Multikinase (IPMK) to generate some forms of inositol tetrakisphosphate (IP4) and all cellular IP5 [23–26]. The 2’ hydroxyl of inositol is lastly phosphorylated by IP5 2-kinase (IPPK) creating IP6 [26]. Loss of IPMK depletes most or all of the cellular IP5 and IP6 [17,23,24,27]. IPPK knockdown or knockout (KO) depletes all cellular IP6 [17,26].

In recent studies, ablation of the gene encoding either kinase was reported to limit HIV-1 production from HEK293T cells transfected with provirus constructs [17,27]. HIV-1 particles produced from transfected IPMK or IPPK KO HEK293T cells were as infectious as virus produced from WT cells suggesting that IP5 and IP6 have little effect on virion maturation [17]. Earlier work showed that HIV-1 mutants encoding substitutions in Gag/CA residues that affect IP6 binding abolish HIV-1 virion infectivity and destabilize the viral capsid [17,28], suggesting that the IP6-binding site in CA may contribute to HIV-1 capsid function. In this study, we sought to define the consequences of IP5 and IP6 depletion on HIV-1 replication in T cells and the specific steps in the replication cycle that are affected.

## Results

### Inositol phosphates are needed for efficient HIV-1 spread in MT-4 cells

**Fig 1:**
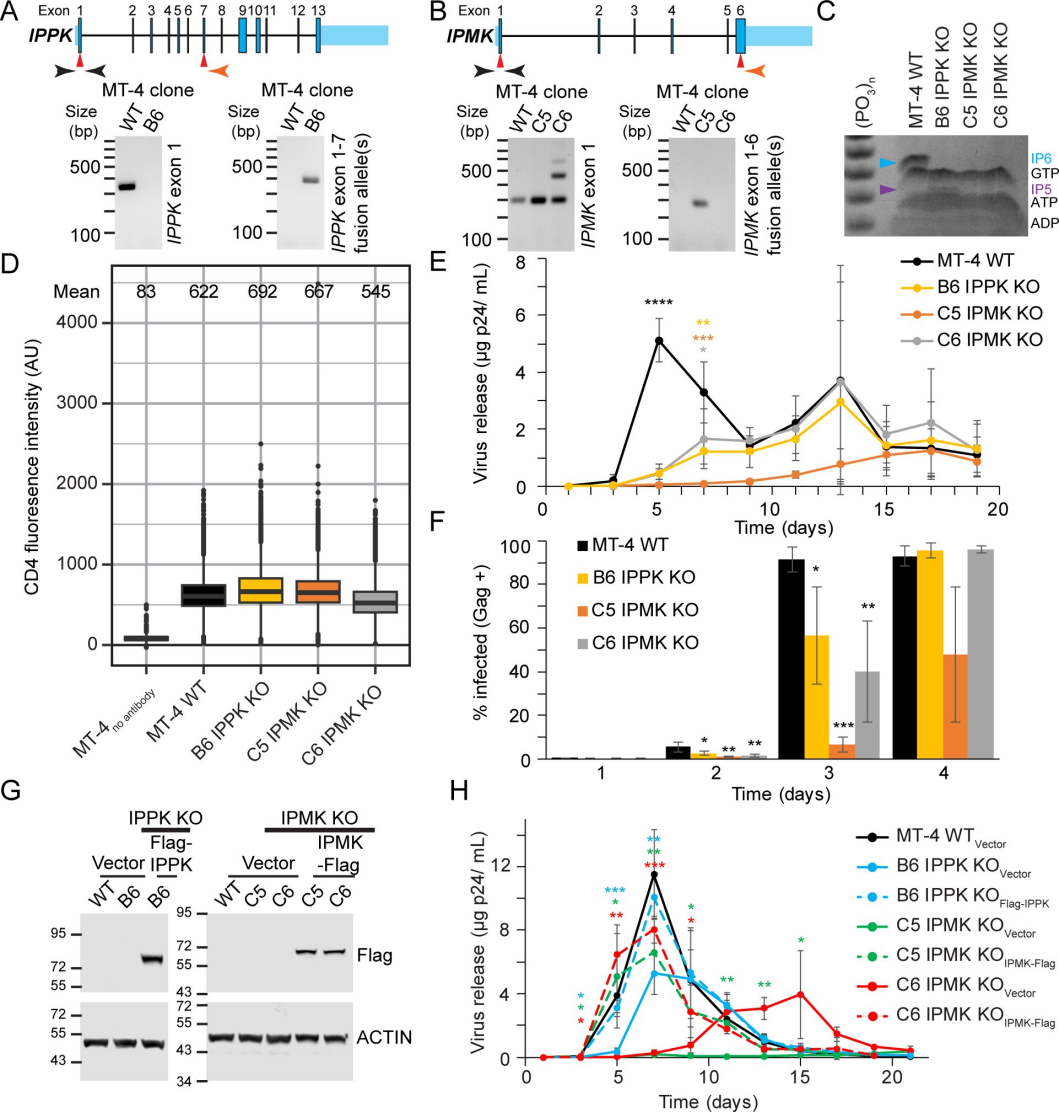
HIV-1 replication in IPPK or IPMK KO MT-4 T cells. A. and B. Diagram of *IPPK* (A) and *IPMK* (B). Top panel: Diagram of *IPPK*. Red arrows indicate approximate Cas9 cleavage site. Genomic PCR primer locations are indicated in black and orange. Primer locations are not exact. Bottom panel: Ethidium bromide stained agarose gel of the indicated, PCR amplified (A) *IPPK* or (B) *IPMK* loci. C. Toluidine blue stained polyacrylamide gel of TiO_2_ enriched phosphorylated compounds. D. Extracellular CD4 expression in the specified cell lines as assessed by flow cytometry. Box shows the 25^th^ to 75^th^ quartiles with the line denoting the median. Outlier points are shown as dots. E. The indicated MT-4 cells (100,000 cells) were infected with 0.1 ng p24 of HIV-1_NL4-3_. Virus release is graphed against time for each MT-4 cell line. F. Bar graph of Gag expression over time for the denoted MT-4 cell lines infected using the 1 ng p24 of HIV-1_NL4-3_ per 100,000 cells. Each bar shows the average of 4 independent experiments. G. Western blot of cell lysates from the designated clones transduced with lentiviral vectors. Protein size markers are shown to the left of the blot in kiloDaltons. H. HIV-1_NL4-3_ release over time using the same amount of p24 per cell as panel (E), but with HIV-1_NL4-3_ concentrated by ultracentrifugation. Each point in (E) and (H) represents the average of 4 independent infections. Asterisks signify significance from WT cells (E) or the respective KO_Vector_ cell line (H). Significance levels: * p<0.05, ** p<0.01, *** p<0.001, **** p<0.0001.

To gain further insight into the physiological relevance of IP5 and IP6 in HIV-1 replication, *IPPK* and *IPMK* were disrupted using CRISPR/Cas9 based gene editing in MT-4 CD4^+^ T cells. PCR amplification of genomic DNA extracted from cloned MT-4 cells electroporated with Cas9/gRNA expression vectors confirmed targeting of *IPPK* and *IPMK* open-reading frames (Fig 1A and 1B). Sequencing of PCR products amplified from the genome of cell clones demonstrated that each clone was genetically null (S1 and S2 Figs). We tested for depletion of IP6 or IP5/IP6 in the *IPPK* or *IPMK* KO clones by polyacrylamide gel electrophoresis (PAGE) [29,30]. IP6 is the predominant inositol phosphate in WT MT-4 cells, and little if any IP5 was detected (Fig 1C). However, in the single *IPPK* KO MT-4 clone we obtained (clone B6), IP5 was elevated and IP6 was undetectable (Fig 1C). By contrast, in *IPMK* KO MT-4 cells, IP6 was depleted, and IP5 remained undetectable (Fig 1C, clones C5 and C6). Thus, disruption of the IPPK and IPMK genes from MT-4 cells depleted IP6. Taking into account previous studies wherein IPMK KO reproducibly lowered IP5 levels, we conclude that *IPMK* disruption likely depleted both IP5 and IP6 [17,23,24,27]. CD4 expression in KO MT-4 clones ranged from 85 to 113% of WT levels (Fig 1D) suggesting that neither cloning nor KO of either protein affected cellular CD4 levels.

We next analyzed the KO MT-4 cells for HIV-1 replication to determine if IP5 or IP6 influence HIV-1 spread. After inoculation with 0.1 ng p24 of HIV-1_NL4-3_, virus release into the culture media was monitored periodically by p24 ELISA. p24 accumulation from WT MT-4 cells peaked at 5 days post infection (dpi), declining shortly thereafter (Fig 1E). Relative to WT cells, HIV-1 replication was delayed in each MT-4 KO clone tested. HIV-1 particles were released at 7 dpi for the B6 IPPK KO and C6 IPMK KO clones, with peak virus production delayed until 13 dpi (Fig 1E). Clone C5 IPMK KO cells displayed the slowest HIV-1 spread, with peak viral loads not occurring until 15 to 17 dpi (Fig 1E). HIV-1 spread remained delayed in all IPPK and IPMK KO clones tested even when the inoculum the was increased by 10- or 180- fold, confirming that the defect in spread was minimally dependent on multiplicity of infection (MOI) (S3A and S3D Fig). We conclude that IP6 promotes HIV-1 spread in MT-4 cells.

To further study HIV-1 spread through the cultures, we analyzed Gag expression by flow cytometry to quantify the fraction of infected cells in the cultures. At 1 dpi, for both MOIs tested, each cell line had roughly the same fraction of Gag-expressing cells indicating that the clones were equally permissive to infection (Figs 1F and S3E). However at days 2 and 3 post infection, the fraction of Gag-expressing cells was higher for the WT compared to the KO cells lines (Fig 1F). Additionally, when the MOI was further increased by 18-fold, the KO clones still had a lesser fraction of Gag-expressing cells at day 2 (S3E Fig). These results demonstrate that IP6 helps HIV-1 spread in culture.

We also complemented the knockout clones by stable transduction with the respective 3xFlag-tagged (Flag) cDNAs and analyzed the effects on HIV-1 replication (Fig 1G). Restoration of expression of IPPK or IPMK resulted in enhancement of replication relative to the respective control vector-transduced KO (KO_Vector_) cells (Fig 1H, compare dashed lines to solid lines). CD4 expression was not affected by KO of either protein in MT-4 cells, and complementation had little effect on CD4 levels relative to the respective KO_Vector_ control (S3H Fig). These results further establish that the slowed HIV-1 spread in IPPK or IPMK KO MT-4 cells is not due to off target effects of CRISPR/Cas9 gene editing or to clonal variation.

### Depletion of IP6 slows HIV-1 spread in T cells

To better resolve the effects of IP5 and IP6 on HIV-1 replication and corroborate the results observed in MT-4 cells, we expanded the KO analysis to include a less HIV-1-permissive CD4^+^ T cell line, CEM. Each gene was genetically ablated using CRISPR/Cas9 technology (S1, S2, S4A and S4B Figs). Further, KO of either gene depleted IP6, confirming loss of the respective kinase (S4C and S4D Fig). The effects of loss of IPPK or IPMK KO on HIV-1 spread were examined to determine whether depletion of IP5 and/or IP6 influences HIV-1 spread. Cultures were inoculated with replication-competent HIV-1 at low multiplicity, and viral accumulation in the media was quantified p24 ELISA. In WT CEM cells, p24 antigen was detected in the media after 6 days and peaked at 9 days post infection (dpi) (S4E and S4F Fig). By contrast, p24 accumulation was delayed for all HIV-1-infected IPPK KO and IPMK KO clones tested (S4E and S4F Fig), suggesting that IP6 might affect virus spread in CEM cells.

**Fig 2:**
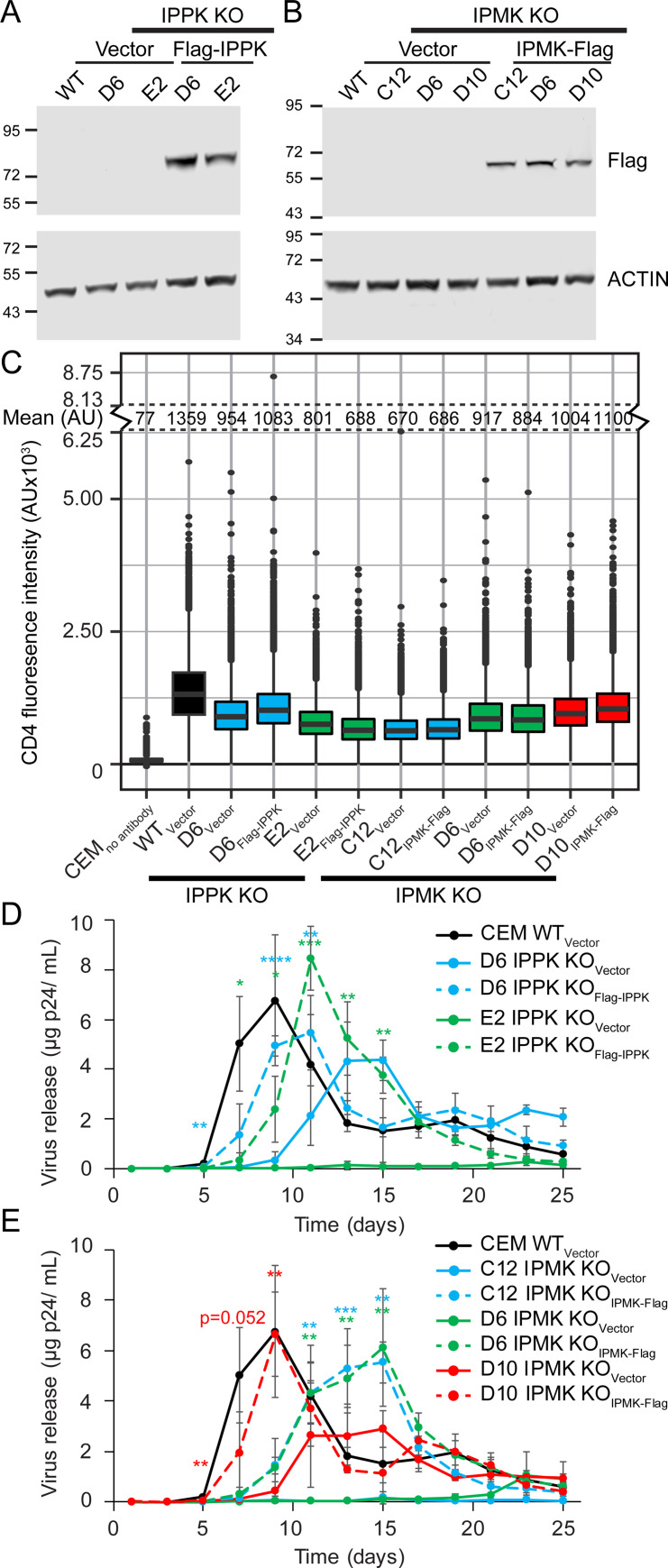
The effect of IPPK or IPMK KO on HIV-1 spread in CEM T cells. A and B. Immunoblot of lysates from the denoted vector only or complemented cell lines. Reference molecular weight markers are shown to the left in kiloDaltons. C. Flow cytometry of extracellular CD4 expression in the denoted CEM cell lines is graphed on a box and whiskers. Box shows the 25^th^ to 75^th^ quartiles with the line denoting the median. Dots show outlier points. D and E. HIV-1 release plotted over time. Each point represents the average of 4 independent infections. Asterisks signify significance from the respective KO_Vector_ cell line. Significance levels: * p<0.05, ** p<0.01, *** p<0.001, **** p<0.0001.

To further characterize the IPPK- or IPMK- negative CEM clones, we quantified the mean florescence intensity (MFI) of cell-surface CD4 expression by flow cytometry. WT cells exhibited broad cell surface CD4 levels (S4G and S4H Fig). CD4 expression varied between WT and KO CEM clones, ranging from 20 to 107% of WT CD4 MFI, which could affect HIV-1 replication (S4G and S4H Fig). To control for differences in CD4 expression and possible off-target CRISPR/Cas9 gene editing, a subset of CEM KO clones were stably complemented with cDNAs expressing the corresponding gene by transduction (Fig 2A and 2B). CD4 expression by flow cytometry was similar between each pair of KO_Vector_ and complemented cell lines (Fig 2C). We assessed the effect of loss of IPPK or IPMK on HIV-1 spread by comparing replication in the respective KO_Vector_ and complemented cell lines. HIV-1 replicated most efficient in WT_Vector_ cells, peaking at day 9 (Fig 2D and 2E, experiments were performed simultaneously). For both IPPK and IPMK KO_Vector_ cell lines, HIV-1 replication peaked at 13 dpi or later in each KO_Vector_ clone (Fig 2D and 2E). Complementation of the clones with the respective gene accelerated HIV-1 replication relative to that observed in the corresponding KO_Vector_ control (Fig 2D and 2E). We conclude that IP6 is required for efficient HIV-1 spread in T cells.

### HIV-1 assembly, but not initial infection, is decreased in MT-4 cells

**Fig 3:**
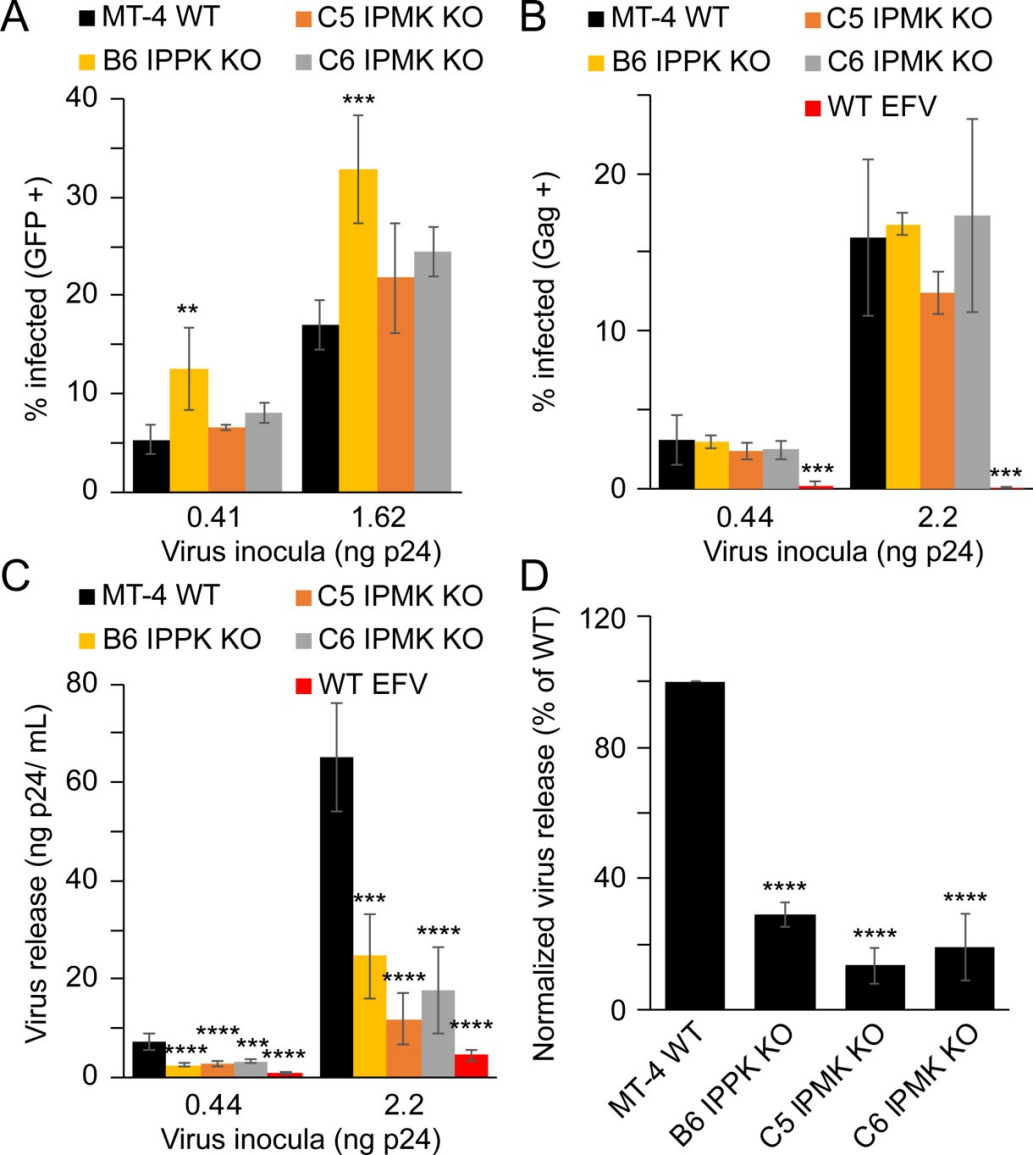
HIV-1 single cycle infection of IPPK or IPMK KO MT-4 cells. A-D. HIV-GFP_VSVg_ single cycle infection (A), HIV-1_VSVg_ single cycle infection (B), virus release from HIV-1_VSVg_-infected cells (C), and single cycle infection normalized p24 release from HIV-1_VSVg_-infected cells (D). Bars in (A-D) represent the average of 3 independent experiments. In panels (A) and (B) single cycle infection measurements are by flow cytometry. Efavirenz is abbreviated as EFV. Asterisks signify significance from WT cells. Significance levels: * p<0.05, ** p<0.01, *** p<0.001, **** p<0.0001.

We next investigated what aspect(s) of viral replication were dependent on IP6. To this end, we initially assayed the permissiveness of the IPPK KO CEM clones to single-cycle infection by VSVg-pseudotyped HIV-1 encoding a GFP reporter gene (HIV-GFP_VSVg_), scoring for infection by flow cytometric analysis of GFP expression. All KO MT-4 cells lines were highly susceptible to HIV-GFP_VSVg_ infection. The B6 IPPK KO clone supported a 2-fold increase in infection relative to WT cells, and IPMK KO cells had an insignificant increase, suggesting that IP6 does not play a significant role in initial HIV-1 infection of MT-4 cells (Fig 3A).

We next corroborated the HIV-GFP_VSVg_ single round infection results and simultaneously monitored HIV-1 particle production to determine whether HIV-1 release differs between WT and IPPK KO clones. To this end, WT and KO MT-4 cells were infected with VSVg-pseudotyped HIV-1 containing a frameshift mutation in the HIV-1 *env* gene (HIV-1_VSVg_), and the cells were separated from the media. Three days after infection cells were fixed and immunostained to test for Gag expression by flow cytometry. The cell-free supernatants were assayed for particle production. The fraction of cells infected was not significantly affected by disruption of the IPPK or IPMK genes (Fig 3B). Thus, the use of CA-immunostaining in flow cytometry accurately reflects HIV-1 infection and Gag expression on a per cell basis.

Concurrent with Gag flow cytometry, HIV-1 particle production from the infected cells was assessed by p24 ELISA of the culture supernatants. IPPK KO and IPMK KO clones produced fewer particles relative to WT cells (Fig 3C). After normalization for the number of infected cells, we determined that infected IPPK KO cells released 29% as much virus as WT cells (Fig 3D). IPMK KO clones exhibited virus production of 13% and 19% of WT for C5 and C6 clones, respectively (Fig 3D). To determine if decreased HIV-1 release from the KO clones resulted from decreased viral gene expression or particle assembly, we analyzed viral gene expression in infected cells using flow cytometry by immunostaining for Gag. Infected IPPK and IPMK knockout cells exhibited greater accumulation of intracellular Gag irrespective of MOI and clone tested (S5A and S5B Fig). Taken together, these data suggest that loss of cellular IP6 decreased HIV-1 particle production owing to an impairment in virus assembly.

### IP5 and IP6 influence early and late events in CEM cells

**Fig 4:**
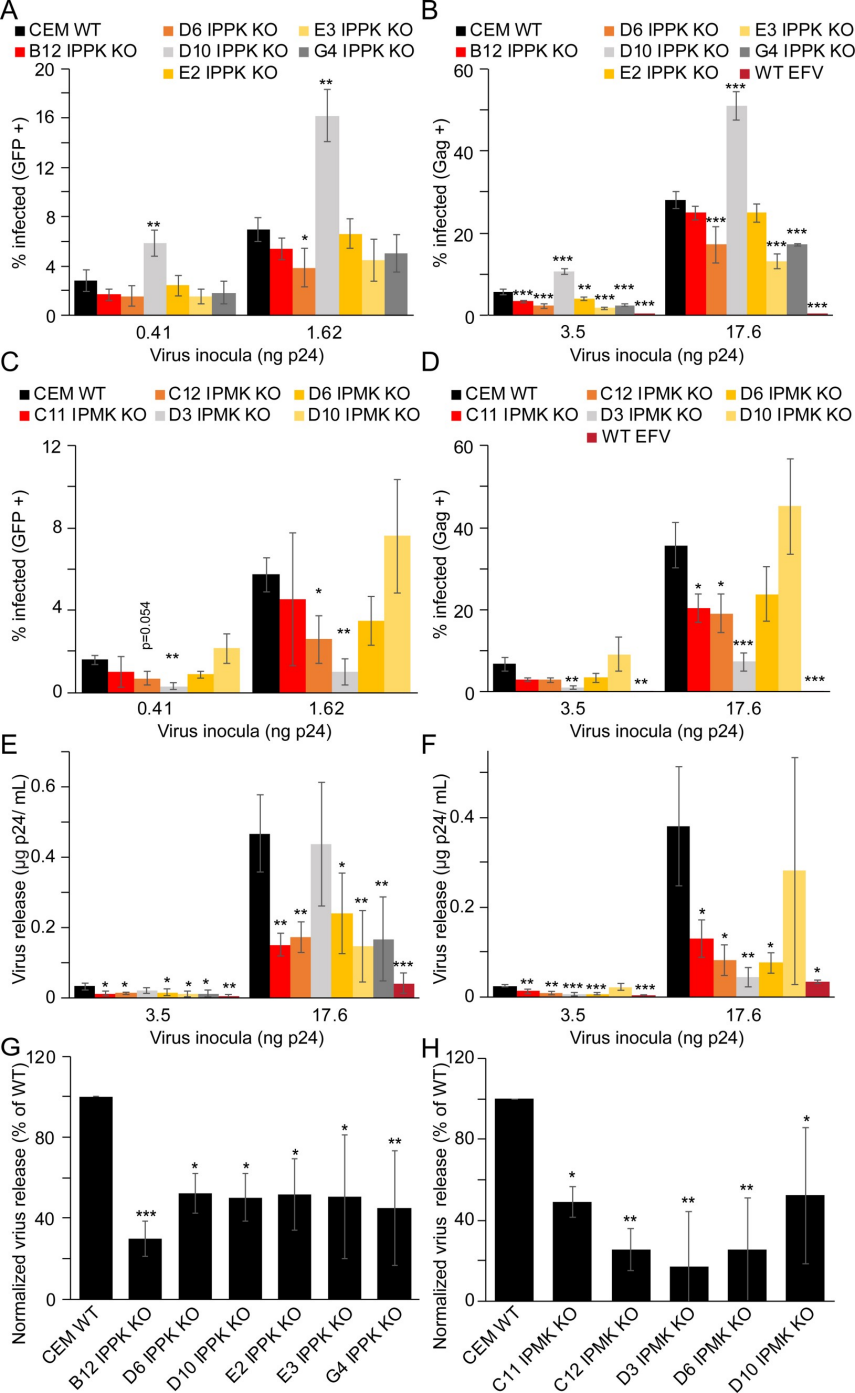
Effect of IPPK or IPMK KO in CEM cells on HIV-1 early and late events. A-H. Bar graphs of HIV-GFP_VSVg_ single round infection (A and C), HIV-1_VSVg_ single round infection (B and D), virus release from HIV-1_VSVg_ infected cells (E and F), and single cycle infection normalized virus release from HIV-1_VSVg_ infected cells (G and H). In all panels, bars represent the average of a minimum of 3 independent experiments. In panels (A) through (D) single cycle infection measurements are by flow cytometry. Efavirenz is abbreviated as EFV. Asterisks signify significance from WT cells. Significance levels: * p<0.05, ** p<0.01, *** p<0.001.

To further define the effect(s) of IP6 on HIV-1, we assayed cell permissiveness to single round infection by HIV-GFP_VSVg_ in our CEM KO cell panel. In CEM IPPK KO cells, the extent of infection varied clonally, with values ranging from ~55 to 77% for clones B12, D6, E3 and G4 relative to infection of WT cells (Fig 4A). HIV-1 infection of the E2 IPPK KO CEM clone was not affected, whereas the D10 IPPK KO clone exhibited increased susceptibility to infection by ~2 fold (Fig 4A). Assays of Gag levels in IPPK KO CEM cells recapitulated the HIV-GFP_VSVg_ results, with all clones except D10 being less permissive to infection than WT cells (Fig 4B). Thus, our results suggest that IP6 depletion in CEM cells tended to lower cell susceptibility to HIV-1 infection.

We also determined whether IP5 promotes to HIV-1 infection of target cells, by challenging the IPMK KO CEM cells with HIV-1. Independent of MOI, IPMK KO decreased the fraction of GFP-expressing, infected cells to 18–72% of WT for IPMK KO clones D3, C11, C12, and D6 (Fig 4C). Conversely, clone D10 was 1.3-fold as permissive as WT cells (Fig 4C). Infection of the CEM IPMK KO clones or WT cells with HIV-1_VSVg_ confirmed these results (Fig 4D). Collectively these results indicate that, in CEM cells, depletion of both IP5 and IP6 decreases HIV-1 permissiveness to a greater extent than depletion of IP6 alone.

To ask whether IPPK or IPMK KO affects virus production in infected CEM cells, we measured p24 release in the culture supernatant of CEM cell lines infected with HIV-1_VSVg_. Except for one clone, all IPPK clones released only 30 to 50% as much virus as WT cells (Fig 4E). The exception, clone D10, exhibited HIV-1 particles at levels comparable to WT (Fig 4E). Ablation of IPMK also decreased the amount of virus released into the medium by all clones except IPMK KO D10 clone, which displayed variable levels of virus release (Fig 4F). Normalization of these values by the fraction of infected cells in the cultures revealed that ablation of IPPK expression reduced HIV-1 production by an average of 70% for clone B12 and by 48 to 56% for clones D6, D10, E2, E3, and G4 (Fig 4G). Independent of IPMK KO clone tested, ablation of IPMK significantly decreased HIV-1 release on a per cell basis, with several clones exhibiting at least a 4-fold reduction in virus production (Fig 4H). Since disruption of IPMK function affected release to a greater extent than ablation of IPPK, we suggest that both IP5 and IP6 contribute to HIV-1 particle production in T cells, or IP5 is able to partially fulfill the role of IP6 in IPPK KO cells.

To test whether the reduced yield of virions was associated with an impairment in HIV-1 assembly or viral gene expression, we quantified Gag levels in HIV_VSVg_-infected cells. We observed that in IPPK KO clones D10 and to a lesser extent D6 intracellular Gag was elevated. In all other IPPK KO CEM clones tested, disruption of IPPK had no effect on intracellular Gag levels (S5C and S5D Fig). In IPPK KO clone D10, Gag expression was particularly high relative to all other clones. Cells with increased HIV-1 gene expression (i.e. those with high amounts of Gag) are more likely to be gated as Gag positive by flow cytometry, perhaps explaining the increased apparent permissiveness of clone D10 to HIV-1. Consistent with the observed defect in Gag assembly or release, Gag levels were increased for all IPMK KO CEM clones, with the exception of clone D3, in which decreased Gag expression was observed (S5E and S5F Fig). The lower HIV-1 gene expression in clone D3 could explain its particularly lower fraction of GFP or Gag positive cells in Fig 4C and 4D. We conclude that disruption of IPPK in CEM cells results in a modest decrease in HIV-1 particle production, which likely contributes to the observed delay in viral spread. In the context of IPMK KO, our results suggest that IP6 contributes to HIV-1 spread by increasing the quantity of virions released from an infected cell, resulting in intracellular Gag retention consistent with an assembly defect.

### Depletion of IP6 lowers progeny virion infectivity

**Fig 5:**
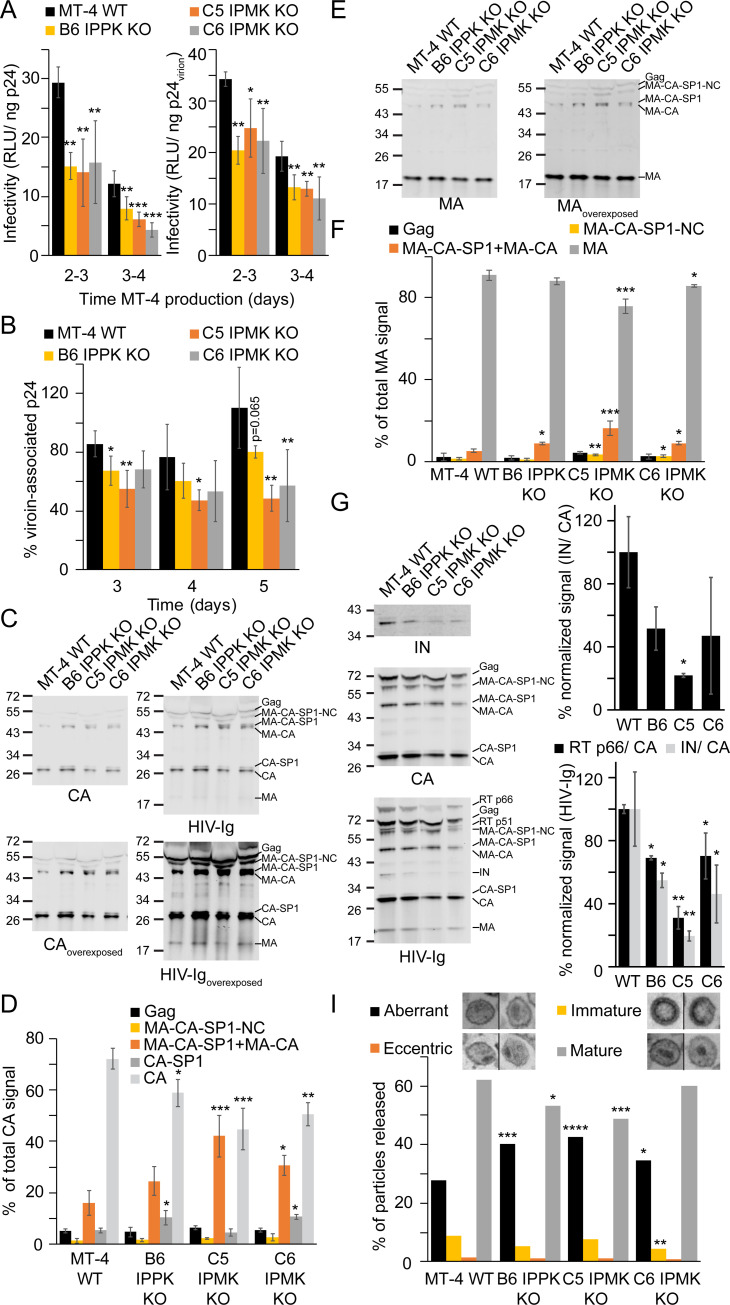
HIV-1 progeny virions from IPPK or IPMK KO MT-4 cells. A. Left panel: infectivity of HIV-1 particles released from the infected WT and KO MT-4 cells expressed as relative light units (RLU) per ng of input p24, assessed by titration on TZM-bl reporter cells. Right panel: same as left panel except values were adjusted to account for differences in virion-associated (i.e. pelletable) p24. B. Fraction of virion-associated p24 at each day post infection. C and E. Immunoblots of HIV-1_NL4-3_ virions from cell-free supernatant probed with the indicated antibodies. Sizes are denoted to the left of the blot in kiloDaltons. D and F. Quantification of immunoblots in (C) and (E), respectively. G. Left panel: Western blot of HIV-1_NL4-3_ particles released from the indicated cell lines pelleted through a 20% sucrose cushion. Sizes to the left of the blot are in kiloDaltons. Right panel: Quantification of the immunoblots described in the left panel. The signal of the indicated protein was normalized for loading using the total CA signal for the respective lane. IN signal using an antibody against IN is quantified in the top graph. The signal corresponding to RT p66 or IN upon probing with HIV-Ig is shown in the bottom graph. H. Morphologies of HIV-1 particles released from the indicated cell lines as determined by thin section electron microscopy. In (A) and (B) each bar represents the average of 4 independent experiments. In (D) and (F) each bar represents the average of 3 independent experiments. In (G) each bar represents the average of 2 independent experiments. Asterisks signify significance from WT cells. Significance levels: * p<0.05, ** p<0.01, *** p<0.001, **** p<0.0001.

In addition to promoting HIV-1 particle assembly, IP5 and IP6 also promote the assembly of recombinant CA protein into conical capsids *in vitro* [11]. Furthermore, HIV-1 mutants containing substitutions in the IP6 binding site in CA are non-infectious [11,17,28]. Therefore, we hypothesized that the virus released from IPPK KO or IPMK KO cells might be less infectious than the virus made in WT cells. To produce quantities of HIV-1 progeny virions for analysis, the WT and KO MT-4 cells were infected with HIV-1_NL4-3_ at a high MOI, and culture media was collected each day following infection. Between days 2 and 4 after inoculation, cells released HIV-1 progeny virions at adequate levels in each MT-4 cell line for single-cycle assays of progeny virion infectivity in TZM-bl reporter cells [31] (S3D Fig). For all KO clones, progeny viruses made during either window of virus production were approximately half as infectious as those released from WT MT-4 cells (Fig 5A, left panel). These results demonstrate that HIV-1 released from IP6 depleted cells is less infectious than virus released from WT cells.

Alterations to progeny HIV-1 infectivity may be caused by defects in the particle and/or in early events. Since HIV-1 assembly or release was affected by KO of IPPK and IPMK, we tested for particle-associated deficiencies. To examine the proportion of HIV-1 free and particle-associated p24 released from infected cells, the p24 content in the initial cell culture supernatants was quantified by ELISA before and after pelleting of virions through a 20% sucrose cushion. The majority of p24 released from WT cells was virion-associated, but the fraction of virion-associated p24 was lower for the KO cell lines (Fig 5B). On average, across all time points WT, B6 IPPK KO, C5 IPMK KO, and C6 IPMK KO cells had 91, 69, 50, and 60% virion-associated p24, respectively, (p < 0.01, relative to WT for all KO cell lines). Thus, IPPK KO and IPMK KO cells released elevated quantities of non-virion-associated p24. Even upon normalization of the infectivity data for differences in virion-associated p24, progeny virions from each KO clone remained less infectious than their WT counterparts (Fig 5A, right panel). Therefore, our results suggest that progeny virions released from IP6 depleted cells have additional defects beyond decreased virion-associated p24.

Because infectivity and virion-associated p24 were both reduced by IPPK or IPMK KO, we next tested if HIV-1 maturation was affected by depletion of IP6. Particles produced by the MT-4 cells were analyzed by western blotting for Gag cleavage efficiency. The majority of the Gag protein in HIV-1_NL4-3_ particles released from WT cells was fully cleaved into CA (Fig 5C). By contrast, particles from IPPK or IPMK KO cells exhibited incompletely cleaved Gag proteins, including a protein migrating below MA-CA-SP1 (Fig 5C and 5D). This band migrated at a size consistent with premature cleavage of SP1 from MA-CA-SP1. It was also detected by probing with HIV-1 Ig and MA antisera demonstrating that the band was the Gag cleavage intermediate MA-CA (Fig 5C and 5E, MA-CA). Compared to virions released form WT cells, virions released from IPPK and IPMK KO cells exhibited decreased quantities of mature CA and MA cleavage products (Fig 5D and 5F). Virions produced by B6 IPPK KO and C6 IPMK KO cells had increased proportions of CA-SP1 and MA-CA-SP1/MA-CA cleavage intermediates relative virus produced in WT cells (Fig 5D and 5F). Particles released from C5 IPMK KO cells had a higher fraction of MA-CA-SP1/MA-CA than particles released from WT MT-4 cells. Collectively, these results suggest that depletion of IP6 results in defects in Gag processing.

We next investigated if the Gag-Pol polyprotein, encompassing each Gag domain, PR, reverse transcriptase (RT) p66, RT p51, and integrase (IN), was differentially incorporated into HIV-1 particles released from IPPK or IPMK KO cells. Virions released from each cell line were concentrated by pelleting through a 20% sucrose cushion to remove non-virion associated proteins, and the resulting pelleted particles were examined by western blotting. Whereas particles produced by WT MT-4 cells efficiently incorporated IN and RT p66, HIV-1 particles released from B6 IPPK KO cells and each IPMK KO clone had lower amounts IN and RT p66 incorporated into the virion (Fig 5G). These data suggest that HIV-1 released from IP6 depleted cells either package lower amounts of Gag-Pol or that Gag-Pol is inefficiently processed by PR upon IP6 depletion.

Impairments in Gag processing can alter particle morphology and inhibit HIV-1 replication [32–36]. To test for evidence of budding defects and alterations to particle morphology upon depletion of IP5 and/or IP6, we examined infected MT-4 cells by thin section transmission electron microscopy at 3 days following infection. All MT-4 cell lines infected with HIV-1_NL4-3_ had appreciable amounts of HIV-1 particles adjacent to the cell membrane, and no virus assembly intermediates could be conclusively identified within any MT-4 cell lines (S6 Fig). WT MT-4 cells frequently had large amounts of HIV-1 particles near the plasma membrane (S6B Fig). Fewer HIV-1 particles were released adjacent to the plasma membrane of cells lacking IPPK or IPMK (S6C–S6E Fig), consistent with an assembly or release defect. We observed no increase in the quantity of incompletely budded or tethered virions to the surface of the KO cells, suggesting that HIV-1 budding is not selectively impaired by IPPK or IPMK disruption.

To test if viral particle morphology was altered by KO of IPMK or IPPK, particles were classified into 4 morphologies [34,35,37]: (1) aberrant particles having a diffuse, ill-defined core with little obvious electron dense material; (2) eccentric particles containing crescent shaped, envelope adjacent electron dense material; (3) immature particles showing an electron dense circle or semicircle corresponding to the immature Gag lattice adjacent to the viral envelope; and (4) mature particles that contain an electron dense conical core (Figs 5H, legend, and S6, arrowheads). Mature particles were detected in association with WT cells, comprising 62% of virions, with aberrant and immature morphologies representing the two next most prominent classes (Fig 5H). Viruses produced by the KO cell lines were predominantly of the mature morphology. However, KO of either kinase increased the proportion of aberrant particles from 27.8% of particles produced in WT cells to 40.3, 42.5 and 34.6% of particles produced in B6 IPPK KO, C5 IPMK KO, and C6 IPMK KO cell lines, respectively (Fig 5H). These results indicate that depletion of cellular IP6 causes a defect in HIV-1 maturation that is manifested as a reduction in virion infectivity.

### Maturation of released virions is altered in cells depleted for IP5 and/or IP6

**Fig 6:**
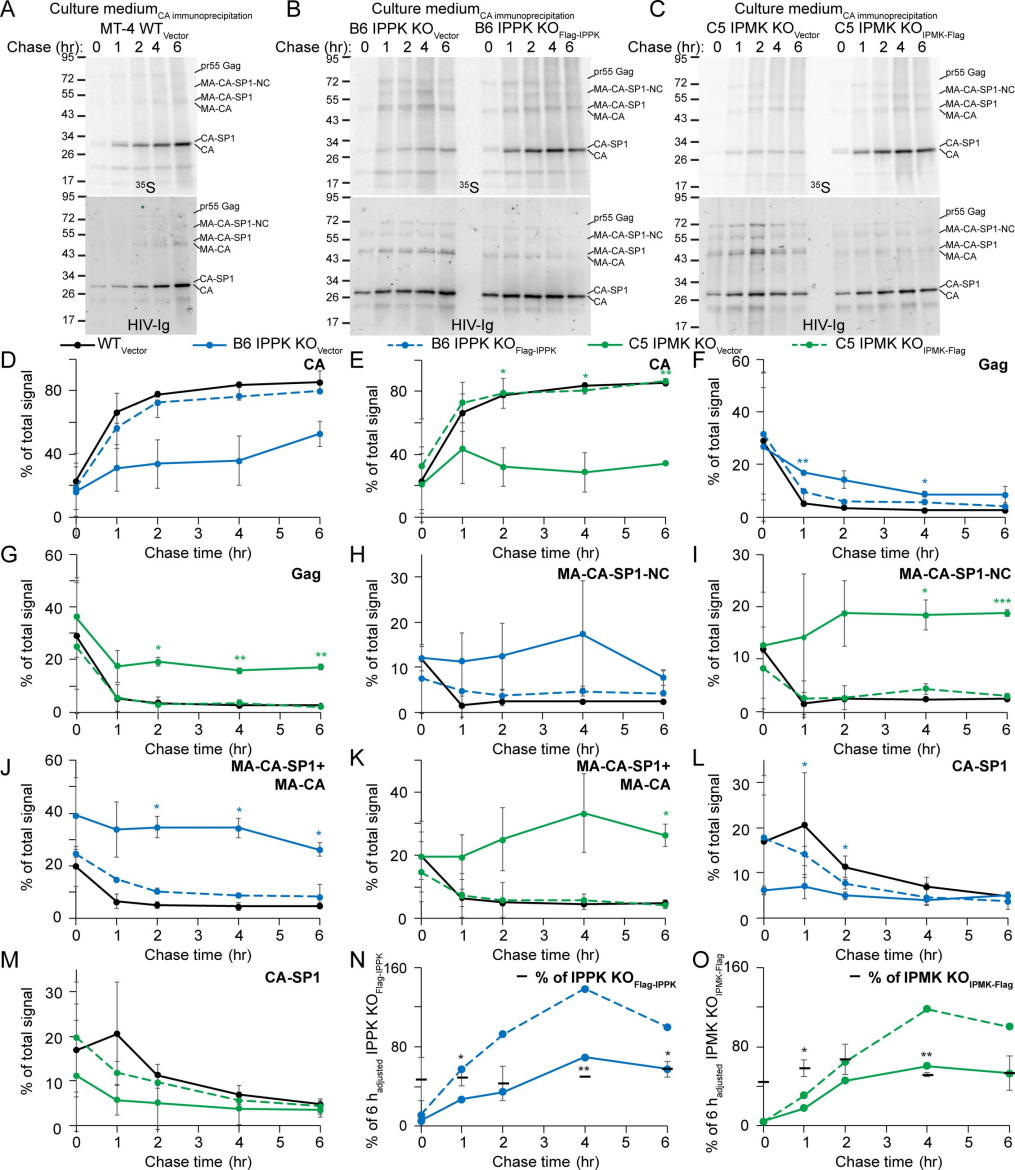
^35^S pulse chase of HIV-1 virions produced in IPMK or IPPK KO cells. A–C. Immunoblots of CA immunoprecipitants from ^35^S labeled progeny virions released from the indicated cell lines. Protein sizes are to the left of each blot in kiloDaltons. Top panel: Phosphorimaging scan. Bottom panel: Blot probed with HIV-Ig. D–O. Quantification of the ^35^S signal from released HIV-1 particles at each chase time point in hours (hr) for CA (D and E), Gag (F and G), MA-CA-SP1-NC (H and I), MA-CA-SP1+MA-CA (J and K), CA-SP1 (L and M), and the adjusted total signal in each lane relative to the respective 6 hour (h) back complemented cell line (N and O). In N and O, values for the KO_Vector_ data points are adjusted by dividing by the transcription/translation efficiency for the respective KO cell line. In panels A–O, HIV-1 particles released into the cell medium were used for CA immunoprecipitation. Points represent the average value from to 2 independent ^35^S pulse/ chase experiments. Significance levels: * p<0.05, ** p<0.01, *** p<0.001.

Owing to the apparent maturation defect associated with virions released from KO cells, we performed pulse-chase analysis of Gag synthesis and cleavage in IP5 and/or IP6-depleted cells using the MT-4 KO cell lines. PR can cleave Gag both prior to and after budding [7]; therefore, at the 0, 1, 2, 4, and 6 hour chase endpoints the media and cells were separated from each other and subsequently lysed, subjected to CA-specific immunoprecipitation, and eluates were immunoblotted. Blots were initially probed with HIV Ig antisera to detect total steady-state CA signal in the immunoprecipitates. By contrast to the WT_Vector_ cell line, in each KO_Vector_ cell line we observed accumulation of a Gag cleavage intermediate corresponding to uncleaved MA-CA and MA-CA-SP1 in immunoprecipitates from both released virions and cellular lysates (Figs 6A and S7A, HIV Ig). Complementation of the KO cells with the respective protein resulted in suppression of the levels of this band (Figs 6B, 6C, S7B and S7C). These results suggest that IP5 and/or IP6 promotes proteolytic Gag processing during maturation.

Quantification of the ^35^S-labeled intracellular Gag cleavage products in the sample showed that cleavage was delayed in B6 IPPK KO_Vector_ cells relative to WT_Vector_ and B6 IPPK KO_Flag-IPPK_ lines (S7D and S7F Fig). The intracellular Gag cleavage rate in C5 IPMK KO_Vector_ cells was variable, yet IPMK complementation stimulated cleavage relative to that in C5 IPMK KO_Vector_ cells (S7G–S7I Fig). Thus, complementation of either protein stimulated intracellular PR cleavage of Gag suggesting that inositol phosphates might affect intracellular Gag processing.

We next tested if Gag processing within released HIV-1 particles is influenced by IP5 and IP6. By contrast to radiolabeled particles released from WT_Vector_ cells which were predominantly comprised of CA, ^35^S-labeled particles released from each KO_Vector_ cell line contained elevated levels of several Gag processing intermediates that were decreased by complementation (Fig 6A–6C, ^35^S). Generation of CA was delayed in particles released from B6 IPPK KO_Vector_ and C5 IPMK KO_Vector_ cells relative to particles released from WT_Vector_ and complimented controls, with CA reaching 52% and 34% of the total signal at 6 h, respectively, compared to the 80–86% of the total signal attained for the controls (Fig 6D and 6E). Consistent with this, processing of Gag and its cleavage products was slowed in each KO_Vector_ cell line, especially for MA-CA-SP1-NC and MA-CA-SP1/MA-CA which displayed little processing over the 6 h chase period relative to control cell lines (Fig 6F–6K). We conclude that IP5 and IP6 stimulate processing of the MA-CA PR cleavage site in Gag, with both metabolites affecting cleavage between SP1-NC as well, albeit to a lesser extent.

To examine the potential effects of IPPK and IPMK disruption on HIV-1 release, we quantified the radiolabeled signal corresponding to Gag and its cleavage products released from infected KO_Vector_ and complemented cell lines. At each timepoint the total amount of radiolabeled protein in CA immune complexes released from KO_Vector_ cells was less than that from the corresponding complemented cell line (Figs 6B, 6C and S8). However, the total intracellular ^35^S signal during the pulse was affected by IPMK KO and to a far lesser extent by IPPK KO (S7B and S7C Fig). Upon normalization to account for differences in HIV-1 transcription and/or Gag translation imparted by IPPK or IPMK KO, HIV-1 release from KO_Vector_ lines into the media remained decreased by ~2-fold compared to release from complemented cells (Fig 6N and 6O). Our results indicate that inositol phosphates affect HIV-1 assembly or release and that this effect is not due to effects on virus transcription or translation imparted by KO of IPPK or IPMK.

## Discussion

**Fig 7:**
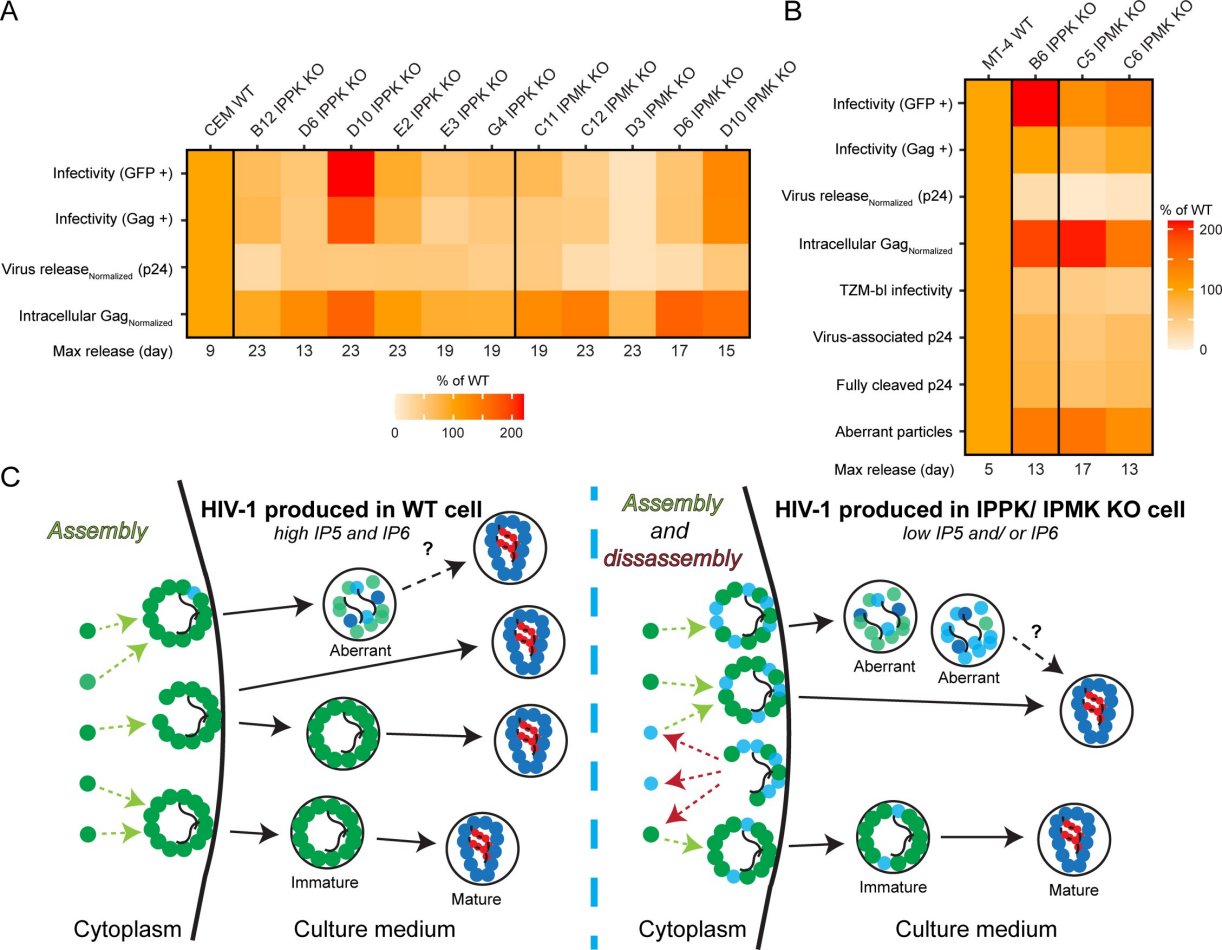
Summary of IPMK or IPPK dependent HIV-1 phenotypes. A–B. Heatmaps summarizing the effects of IPPK or IPMK deficiency on HIV-1 replication in (A) CEM or (B) MT-4 cells. For experiments where multiple HIV-1 inocula were used, values shown are the average of the percent of WT. C. Model of the effect of depletion of IP5 and/or IP6. Green circles represent Gag. Teal circles represent MA-CA-SP1/MA-CA. Red circles represent NC. Squiggly lines represent viral RNA. Solid black lines with arrows depict release and changes in virion morphology. Dashed arrows portray assembly (light green) or disassembly (dark red) of Gag or Gag processing intermediates.

In this study we examined the effects of IPPK and IPMK gene disruption on HIV-1 replication in T cells. Our results demonstrate that the inositol kinases IPPK and IPMK influence HIV-1 spread via multiple mechanisms that affect the quantity and quality of HIV-1 particles released from cells (Fig 7). The effects observed were specific with the same impairments in HIV-1 spread, viral assembly/ release, and Gag processing detected in all IPPK and IPMK KO clones tested (Figs 1, 2, 3D, 4G, 4H, 5, 6, 7A and 7B). Depletion of IP6 was deleterious to HIV-1 assembly or release (Figs 3D, 4G, 4H, 6N, 6O, S5 and S8). However when studies of *in vitro* Gag assembly are considered [11,14] along with the similar efficiencies of HIV-1 budding observed between KO and WT cells by EM (S6 Fig), we conclude that the decrease in HIV-1 release is due to inefficient intracellular Gag assembly in the absence of IP6 rather than a release defect (Fig 7C, Cytoplasm).

Disruption of IPMK was more deleterious to aspects of HIV-1 replication than KO of IPPK. Specifically, viral spread, release, Gag processing, and virion morphology were affected more (Figs 1–6,7A, 7B and S5). Because IPPK KO cells accumulate IP5 (Fig 1C), these data suggest that in the absence of IP6, IP5 promotes HIV-1 replication. Lower inositol phosphates, including IP4 and IP5, can promote Gag assembly *in vitro* [11]. Moreover, IP5 substituted for IP6 in progeny virions released from provirus transfected IPPK KO HEK293T cells [17], and IPPK KO cells make appreciable amounts of HIV-1 progeny (Figs 3D,4G and 7N). HIV-1 was still appreciably released upon depletion of IP5 and IP6 (Figs 3D, 4H, 7O, and [17]). Therefore, we suggest Gag assembly within the cell may exploit additional inositol phosphates as cofactors.

Alternatively, the small amount of IP6 that persists in IPMK KO cells might be sufficient to facilitate Gag assembly at the low level observed in our study (S4D Fig). Accordingly, residual IP6 in IPMK KO HEK293T cells was previously identified to be incorporated into released HIV-1 particles, suggesting that even at low concentrations IP6 can drive intracellular Gag assembly [17]. Depletion of IP5 and residual IP6 via expression of the 3’ inositol phosphatase MINPP1 in IPPK KO HEK293FT cells resulted in release of few to none HIV-1 particles, suggesting that only IP5 and IP6 are able to facilitate Gag assembly *in cellulo* [27]. However, caution should be taken when interpreting each of these results. The HEK293T, and HEK293T derived HEK293FT cells, used for KO of IPPK or IPMK in references 17 and 27 express viral oncogenes that manipulate steady state inositol phosphates levels [38,39]. Second, ectopic expression of MINPP1 in IPPK KO HEK293FT cells was cytotoxic, making it difficult to interpret the results using this method [27].

Our data using IPPK and IPMK KO CD4+ T cell lines might not support the notion that residual IP6 is incorporated into released virions either: in MT-4 cells IPMK KO caused a more pronounced maturation defect in released particles than did IPPK KO (Figs 6 and S7). Taking all of this into account, it is also possible that other HIV-1 assembly factors may exist, and more experimentation will be required to precisely quantify the spectrum of inositol phosphates encapsidated within virions released from IPPK or IPMK KO CD4^+^ T cells. Identification of metabolites that bind Gag when IP5 and IP6 are depleted will be of interest.

Our results contrast with a recent study examining HIV-1 released from provirus-transfected HEK293T cells, in which IPPK or IPMK disruption did not affect virus infectivity [17]. By contrast, we observed that progeny virions budded from IPPK or IPMK KO cells were less infectious and contained particle-associated defects (Figs 5 and 6). This difference could be a consequence of differences in the experimental systems (i.e. transfection versus infection, differences in time of HIV-1 progeny virion harvest, and quantitation methods) or differences between the cell type used (i.e. HEK293T versus CD4^+^ T cells). CD4^+^ T cells are the physiological target of HIV-1 and depletion of this cell type by HIV-1 causes acquired immunodeficiency syndrome.

### How do inositol phosphates promote efficient Gag processing?

Before HIV-1 is released from IPPK or IPMK KO cells, the depleted IP6 presents an obstacle (Fig 7C). In the presence of IP6, the SP1 six-helix bundle is stabilized [11], thus Gag assembles into immature particles begins to bud, is cleaved by PR, and the particle is released containing a mature core (Fig 7, WT cell) [40]. In the absence of IP6, Gag assembly is inefficient *in vitro* [11] and *in cellulo* (Figs 3D, 4G, 4H, 6N, 6O and S8) [17]. Consistent with this, molecular dynamics simulations from the Perilla lab suggest that if IP6 fails to bind the Gag SP1 six-helix bundle, the Gag lattice is unstable [11]. Therefore, in the absence of IP6 and perhaps IP5, Gag may transiently form partial lattices, and activate PR, but may fail to completely assemble, leaving partially cleaved, intracellular Gag intermediates. Some of these partially cleaved Gag molecules may co-assemble with budding particles reducing their infectivity. This model underscores the importance of temporal regulation of Gag cleavage during HIV-1 maturation and might explain the decreased amount of IN and RT p66 incorporated into virions (Fig 5G).

Morphologically aberrant HIV-1 virions containing partially cleaved Gag were previously observed to be released from cells treated with suboptimal concentrations of the PR inhibitor lopinavir (LPV) [35]. The suboptimally LPV-treated virions have quantitatively similar infectivity, MA-CA-SP1/MA-CA levels, and morphological defects to HIV-1 particles released from IPPK/IPMK KO cells [35]. Thus, we suggest that depletion of IP6 partially phenocopies suboptimal LPV concentrations by increasing the abundance of Gag processing intermediates inside viral particles leading to the creation of aberrant particles (Figs 5,6 and 7C). Although it is tempting to speculate that IP6 directly affects PR activity, *in vitro* cleavage of immature Gag assemblies is actually slowed by IP6 [15]. Therefore, a more plausible explanation for the observed maturation defect in particles released from IP6 depleted cells is that lower amounts of Gag-Pol, and consequentially PR, are packaged into the virion thereby slowing maturation (Fig 5G). Notably, we were unable to detect uncleaved Gag-Pol in released virions, thus it remains possible that, like Gag, Gag-Pol is more slowly processed within HIV-1 particles released from IP6 depleted cells. Further, immature lattice formation in itself might also be needed for PR to cleave the MA-CA (or CA-SP1) junction, and that without IP6 stabilization, insufficient active PR dimers are formed, slowing maturation [7].

### How does IP6 binding to CA enhance HIV-1 infectivity?

The majority of viral particles released from IPPK or IPMK KO MT-4 cells displayed the mature core morphology (i.e. an electron dense conical core) (Fig 5H) suggesting that inositol phosphate binding is not a prerequisite for CA assembly *in virio*. Supporting this view, studies of HIV-1 virions bearing Arg18 substitutions suggest that these mutations affect capsid stability, yet the virions still contain mature cores [18,28]. Further, several Arg18 mutant CA proteins, including R18A and R18A/N21A, preferentially form spheres and cones *in vitro* rather than the tubes polymerized by WT CA, indicating that Arg18 mutant CA proteins are competent for assembly [41]. Thus, mature core morphology seems to be disconnected from capsid stability. Virions lacking IP6 owing to mutation of residues needed for IP6 binding during Gag assembly have unstable capsids [17], and IP6 increases capsid stability *in vitro* [12]. Collectively, these observations suggest that the IP6/CA interaction may be dispensable for CA assembly *in virio*, but inositol phosphate binding to CA may enhance capsid stability, thus promoting reverse transcription.

Addition of inositol phosphates affects endogenous reverse transcription reactions, making them resistant to DNAseI, presumably by stabilizing the viral capsid [12]. Recently, efficient HIV-1 reverse transcription has been fully reconstituted *in vitro* in the absence of cellular extracts [19,20]. These *in vitro* reverse transcription reactions require IP6 stabilization of the capsid enable reverse transcriptase to polymerize full length viral DNA [19,20]. Thus, IP6 might stabilize the viral capsid during early steps of infection. However, our results in IPPK and IPMK KO MT-4 cells, and results of others using HEK293T cells, do not support a major role of target cell inositol phosphates in early steps of infection (Figs 1F, 3, 7B, S3E, and [17,27]). By contrast, in CEM cells, we observed that HIV-1 single cycle infection was typically decreased in most IPPK KO and IPMK KO clones, with IPMK KO being more deleterious to single cycle infection (Figs 4A–4D and 7A). Thus, the IP6 stabilization of the capsid appears to be cell line dependent, possibly due to other capsid stabilizing or destabilizing factors being present in different cell types. Our results provide new insight into how HIV-1 Gag and CA commandeer inositol phosphates to enhance virus spread by directing aspects of immature virion assembly and subsequent progeny virion infectivity.

## Materials and methods

### Cell culture and virus production

TZM-bl [31], CEM (CEM-T4), and MT-4 cells were purchased from the NIH AIDS reagent program. HEK293T cells were acquired from American Type Culture Collection. HEK293T and TZM-bl cells were maintained in complete DMEM: DMEM (Corning, 10-013-CV), supplemented with 100 IU/mL penicillin, 100μg/mL streptomycin, and 10% (vol/vol) fetal bovine serum (FBS). MT-4 and CEM cells were maintained in complete RPMI: RPMI-1640 (Corning, 10-040-CV) supplemented with 100 IU/mL penicillin, 100μg/ mL streptomycin, and 10% (vol/ vol) FBS. Cell lines were maintained in a humidified 37°C incubator with 5% CO_2_.

HIV-1 and the lentiviruses used for complementation were produced by calcium phosphate transfection of HEK293T cells with the following plasmid combinations. To make HIV-GFP_VSVg_, pNL4-3Δ*env*Δ*nef*-GFP was co-transfected with pCMV-VSVg [42]. HIV-1_NL4-3_ was generated by transfecting pNL4-3 [43]. HIV-1_VSVg_ was produced by co-transfection of pCMV-VSVg and pNL4-3Δ*env*. 3x-Flag-IPPK (Flag-IPPK) or IPMK-3x-Flag (IPMK-Flag) expressing lentiviruses were created by transfection of the respective pLenti-EF1-IRES-blasticidin construct with pCMV-VSVg and psPAX2. All HIV-1 viruses used in this study were concentrated by ultracentrifugation at 154,224 x g from 3 hours at 4°C in a SW32 rotor with the exception of the viruses used in Figs 1E, 5A, S3A, S4E and S4F. For all viruses used in this study, viral titer was quantified by p24 ELISA [44] and flow cytometry for Gag or GFP expression.

### Plasmid cloning and CRISPR/Cas9 KO

All oligonucleotide sequences were generated by IDT and sequences are annotated in S1 Table. Plasmid sequences were verified by Sanger sequencing (Genewiz). pX458 constructs were generated by annealing the oligonucleotides indicted in S1 Table and ligating them into the *Bbs*I site of pX458 [45]. Successive rounds of nested PCR were used to create Flag-IPPK and IPMK-Flag (S1 Table). PCR products were Gibson cloned into *EcoR*I digested pLenti-EF1-IRES-Blasticidin to create pLenti-EF1-3x-Flag-IPPK-IRES-Blast and pLenti-EF1-IPMK-3x-Flag-IRES-Blast vectors using the NEBuilder HiFi DNA Assembly kit (New England BioLabs).

For CRISPR/Cas9 mediated gene KO, MT-4 or CEM CD4^+^ T cells were electroporated using the Neon Transfection System to deliver pX458 based Cas9/gRNA expression plasmids into the cell (Thermo). A mixture containing pX458-IPPK exon 1 and pX458-IPPK exon 7 were electroporated to KO *IPPK*. To KO *IPMK*, pX458-IPMK exon 1 and pX458-IPMK exon 6 plasmids were simultaneously electroporated. Following a 2 day recovery from electroporation, GFP positive cells were sorted by the Vanderbilt Flow Cytometry Shared Resource core at one cell per well into a 96 well plate using a BD FACS Aria III. Upon clonal outgrowth, genomic DNA was extracted using the Quick-DNA Miniprep Kit (Zymo Research, D3024) and subjected to PCR using Q5 DNA polymerase. Clones were screened for genetic KO by PCR amplification of the first exon and sequences predicted to be deleted upon DNA repair (Figs 1A, 1B, S4A and S4B). The *IPPK* exon 1 gRNA locus was amplified using *IPPK* exon 1 gPCR F and *IPPK* exon 1 gPCR R primers, whereas *IPPK* fusion allele(s) were amplified using *IPPK* exon 1 gPCR F and *IPPK* exon 7 gPCR R primers (S1 Table). *IPMK* exon 1 gPCR F and *IPMK* exon 1 gPCR R primers were used to amplify the exon 1 gRNA locus of *IPMK* (S1 Table). *IPMK* fusion allele(s) were amplified using the primer pair: *IPMK* exon 1 gPCR F and *IPMK* exon 6 gPCR R (S1 Table). PCR products were electrophoresed through 1.2% 1x Tris Acetate EDTA agarose gels in the presence of 0.5 μg/ mL Ethidium Bromide. For Sanger sequencing, PCR products were excised from the agarose gel, purified, and sequenced by Genewiz (S1 and S2 Figs and Sanger sequencing chromatograms in S1 File). *De novo* sequence alignment of DNA sequences from *IPPK* or *IPMK* alleles was performed using Clustal Omega (EMBL) or CLC Genomics Workbench (Qiagen).

### Inositol phosphate TiO_2_ enrichment and separation by native PAGE

Inositol phosphates were purified by TiO_2_ purification as previously described [46]. Briefly, 8x10^7^ cells were grown, pelleted, resuspended in 1.4 mL of PBS, and snap frozen in liquid N_2_. Prior to freezing a 40 μL out of the 1.4 mL PBS-resuspended sample was lysed, and protein was extracted [47]. Extracted protein was quantified by BCA assay (Thermo Scientific) to normalize for cellular input prior loading the PAGE gel. For inositol phosphate extraction, cells were resuspended in 1M perchloric acid and incubated for 15 minutes on ice with frequent agitation. Insoluble materials were pelleted at 18,000 x g for 5 min at 4°C. To bind inositol phosphates to the beads, the supernatant was transferred to a tube containing 4 mg of TiO_2_ beads and incubated at 4°C for 20 minutes on a rotator. Beads were pelleted, washed two times with 1M perchloric acid, and then eluted twice in 200 μL of 2.8% ammonium hydroxide. Eluate was concentrated to 0 to 20 μL using a SC110 Speedvac concentrator. Concentrated elute was tested to confirm that the pH < 8.0, and then eluate was brought up to 60 μL in 10 mM Tris 8.0.

Native Tris Borate EDTA (TBE) PAGE was previously described [29,48]. In brief, for each sample a TiO_2_ eluate equivalent to 3.48 mg of protein were loaded per well onto 35% Tris Borate EDTA PAGE gel. 40 μg of (PO_3_)_n_ ladder (Sodium polyphosphates, SO169, Spectrum Chemical Manufacturing Group) was loaded in one well of each gel to ensure proper resolution. IP5 and IP6 standards were purchased from Cayman Chemical Company (10008453) and TCI (Phytic acid, P0409), respectively. The gel was run at 520 V until the orange G loading dye was 8 cm from the bottom of the gel and subsequently stained for 30 minutes in a solution of 0.1% (wt/ vol) Toluidine Blue, 20% methanol, and 2% glycerol [30]. After destaining the gel for 30 minutes in 20% methanol and 2% glycerol, the destained gel was imaged using a Nikon CoolPix L110 digital camera.

### HIV-1 and retrovirus infections

To infect CEM or MT-4 cells with HIV-GFP_VSVg_, 100,000 cells were plated into a 24 well plate and incubated with 0.41/1.62 or 0.44/2.2 ng p24 per well, respectively. At 2 dpi, HIV-GFP_VSVg_ infected cells were fixed overnight in 2% paraformaldehyde. To test for Gag expression and virus release, 300,000 CEM or MT-4 cells were infected with 3.5/17.6 or 0.44/2.2 ng p24 of HIV-1_VSVg_, respectively. At day 3 day the HIV-1_VSVg_-infected cells were pelleted, and the supernatant was used for p24 ELISA [44]. HIV-1_VSVg_-infected cells were fixed in 2% paraformaldehyde for subsequent processing for flow cytometry.

For spreading HIV-1_NL4-3_ infections, 1 ng p24 of virus was used to infect 100,000 CEM cells. For MT-4 infections with HIV-1_NL4-3_, 0.1 (Fig 1E and 1H) or 1 (S3A Fig) ng p24 of virus was used to infect 100,000 cells. In spreading HIV-1_NL4-3_ infections monitoring virus release (excluding S3D Fig), half of the virus containing media was removed (and replaced) every other day starting at day one. The collected virus containing supernatants were assayed for p24 release by ELISA [44]. In S3D and S3E Fig, 180ng p24 of HIV_NL4-3_ was used to infect 1,000,000 MT-4 cells. Supernatants in S3D Fig were processed as described in the virus-associated, pelletable p24 subsection and used for infection as described in TZM-bl infectivity assay subsection.

For large scale infections where Gag expression was used to track viral spread, 1,000,000 MT-4 cells were infected with 10 ng (Fig 1F) or 180 ng (S3E Fig) p24 of HIV-1_NL4-3_. In Figs 1F and S3E, at 1 dpi cells were pelleted, washed one time in PBS, and resuspended in fresh complete RPMI. At day 2 until the endpoint of the experiment, cells were pelleted each day and 80% of the media was collected and replaced with fresh media. In Fig 1F a quarter, third, half or all the cells were fixed in 2% paraformaldehyde at days 1, 2, 3, or 4, respectively. At each timepoint in S3E Fig, a third, half, and all the cells were fixed in 2% paraformaldehyde at days 1, 2, and 3, respectively. Cells were processed for Gag immunostaining as described in the flow cytometry subsection.

To create vector and Flag-IPPK/ IPMK-Flag back complemented MT-4 or CEM cells, parental clones were transduced with either 2.3 x 10^−5^ or 1.2 x 10^−4^ ng p24 per cell, respectively, of empty vector, Flag-IPPK, or IPMK-Flag lentivirus. Blasticidin resistant cells were selected for and subsequently maintained in 15 or 12.5 μg/ mL blasticidin (Invivogen) for respective CEM and MT-4 cells.

For each MT-4 cell line to be infected for ^35^S labeling, 25,000,000 cells were infected with 688 ng p24 of HIV-1_VSVg_. Infections progressed until 2 dpi when the cells were processed for pulse labeling as described in the methionine/ cystine pulse chase and CA immunoprecipitation subsection.

### p24 ELISA, immunostaining, and flow cytometry

p24 ELISA was as previously described [44]. To normalize for differences in single cycle infection as per Figs 3D, 4G and 4H, the percentage of WT release and infection for each clone was calculated. Then, for each cell line the percentage of WT p24 was divided by the percentage of WT infection to calculate the normalized p24 release.

For flow cytometry experiments, fixed cells were washed in flow-wash buffer (PBS containing 1% BSA) to remove residual paraformaldehyde and permeabilized in 0.25% triton X 100 for 5 min. For CD4 immunostatining, the permeabilization step was omitted. Following three washes in flow-wash buffer, cells were incubated with mouse anti-CA (183-H12-5C, NIH AIDS Repository) at 13.3 ng/ μL or mouse anti-CD4 (RPA-T4) at 2.5 ng/ μL for 1 hour (h) at room temperature. Unbound primary antibody was wash away with 3 washes of flow-wash buffer, and cells were incubated for 1 h at room temperature with goat anti-mouse conjugated to AlexaFluor 514 (A-31555, Invitrogen) at 4 ng/ μL. Cells were washed 3 more times in flow-wash buffer, and flow cytometry was performed using a BD FACS Canto II. For each flow cytometry run, when possible, 20,000 cells were counted per tube. Data was analyzed using the FlowCore R package using custom R scripts or FlowJo 10.1.

### Virus-associated, pelletable p24

At 1 dpi, all the media was removed and the cells were washed one time in PBS. Cells were pelleted, and 800 μL out of 1 mL of the media was collected (and replaced) at 2, 3, 4, and 5 dpi. Prior to ultracentrifugation, 100 μL was removed. 20 μL of this was used as input for p24 ELISA to calculate the total p24 concentration prior to pelleting. The rest of the 100 μL pre-centrifugation input was also used to infect TZM-bl cells (Fig 5A) or for immunoblotting (Fig 5C and 5E). The remaining 700 μL of cell-free virus supernatant was layered on top of 200 μL of 20% (wt/ vol) sucrose (Fisher Scientific) and pelleted at 4°C for 30 minutes at 45,000 rpm in a Beckman TLA45 rotor. The supernatant was removed using a pipet, and the pellet was dissolved in 250 μL of p24 lysis buffer (PBS, 10% donor calf serum, and 0.5% Triton X 100). The amount of pelleted p24 was quantified by ELISA. Percent virion-associated p24 is equal to total pelleted p24 divided by the total p24 in the culture supernatant. In Fig 5G, 800 μL of culture media was pelleted as described above, and the pellet was resuspended in 40 μL of SDS-PAGE loading buffer (31 mM Tris pH 6.8, 1% SDS, 7.5% glycerol, 0.004% bromophenol blue).

### TZM-bl infectivity assay

Prior to infection, virus produced by WT and KO MT-4 cells was quantified for p24 content by ELISA. 10,000 TZM-bl cells were plated in each well of a 96 well plate one day prior to infection. The day of infection TZM-bl cells were inoculated with 0, 0.08, 0.16, 0.31, 0.63, 1.25, 2.5, or 5 ng p24 of HIV-1_NL4-3_ produced in WT MT-4 cells or one of the KO clones. Cells were incubated with virus for 18 h when 1 μM EFV (NIH AIDS Repository, 4624) was added to the media to inhibit viral spread. At 48 hpi, media was removed, cells were washed one time with PBS, and lysed in 1x TBS (50 mM Tris-HCl pH 8.0, 130 mM NaCl, 10 mM KCl, 5 mM MgCl_2_) containing 0.5% triton X 100 for 5 minutes. Luciferase assays were performed as described in ref [47] on a Lmax luminometer (Molecular Devices). At each dose of HIV-1 virus, the RLU/ ng p24 was calculated. To normalize TZM-bl infectivity data for differences in incoming virion-associated p24 between viruses (Fig 5A, left panel), each infectivity value was divided by the fraction of virion-associated p24. All viral doses displayed a linear increase in luciferase activity and were included in the experimental average RLU/ ng p24 infectivity calculation. The average infectivity for four independent experiments was calculated and graphed in Fig 5A.

### Electron microscopy

10^6^ WT, B6 IPPK KO, C5 IPMK KO, or C6 IPMK KO MT-4 cells were infected with 180 ng p24 of HIV-1_NL4-3_ for 24 h. Every 24 h, cells were pelleted at 800 x g and resuspended in fresh complete RPMI 1640 media. At 3 dpi, cells were washed in 0.1 M sodium cacodylate buffer at pH 7.4 and fixed in 2.5% glutaraldehyde in 0.1 M sodium cacodylate buffer for 1 h at room temperature prior to storage at 4°C. Secondary fixation in osmium tetroxide, embedding, ultrathin sectioning, and uranyl acetate staining were as described in ref [49] and performed by the Cell Imaging Shared Resource core at Vanderbilt University. Electron micrographs were taken using a FEI T12 electron microscope equipped with an AMT CCD camera. For categorization of virion morphology, greater than 250 virions were characterized per cell line at a magnification of 25,000 X or greater.

### ^35^S methionine/ cystine pulse chase and CA immunoprecipitation

Labeling of cellular proteins with ^35^S was performed as previously described [50,51]. Briefly, at 2 dpi, cells were washed two times in PBS and incubated for 30 minutes in starvation medium (methionine- and cystine-free DMEM (Corning, 17-204-CI), 10% dialyzed FBS, 25mM HEPES pH 7.4, and 2 mM L-glutamine). Following pelleting 500xg for 5 minutes, the cells were incubated with labeling medium (methionine- and cystine-free DMEM, 10% dialyzed FBS, 25mM HEPES pH 7.4, 2 mM L-glutamine, and 0.5 mCi/ mL ^35^S EasyTag EXPRESS^35^S Protein Labeling Mix (Perkin Elmer, NEG772002MC)) for 30 minutes. Next, cells were washed one (experiment 1) or two (experiment 2, once with PBS and once with chasing medium) times with 2.5 mL of chasing medium (methionine- and cystine-free DMEM, 10% FBS, 25mM HEPES pH 7.4, 2 mM L-glutamine, and 2 mM L-methionine) per cell line to remove residue ^35^S label from the cells and culture medium. A 500 μL aliquot (~5,000,000 cells) was removed from the chase medium wash to collect the 0 h chase sample. Cells were then pelleted and resuspended in 2.0 mL of chase medium and incubated at 37°C for 1, 2, 4, and 6 h, at which point 500 μL of culture were collected. Cells were separated from virus particle-containing medium by centrifuging at 500xg for 5 min at 4°C. Cell medium was immediately frozen at -80°C until the time of processing for CA immunoprecipitation. The cell pellet was further washed twice more with ice cold PBS prior to flash freezing in liquid N_2_ and storage at -80°C.

Cells were lysed for immunoprecipitation in immunoprecipitation lysis buffer (20 mM Tris pH 8.0, 150 mM NaCl, 1% Triton X-100, 0.1% SDS, 1 mM EDTA, and 1x cOmplete EDTA protease inhibitor cocktail (Roche)) for 30 minutes on ice. Virus-containing culture media was lysed using by adding one volume of 2x immunoprecipitation lysis buffer for each volume of medium. Prior to immunoprecipitation, lysates were pelleted at 17,500xg for 10 min at 4°C, and the cellular lysate was quantified by Pierce BCA protein assay kit (Thermo).

For each immunoprecipitation, 10μL of Protein A/G PLUS agarose slurry (Santa Cruz Biotechnology, sc-2003) was pre-bound to 5 μg of mouse anti-CA antibody (183-H12-5C, NIH AIDS Repository, 1513) for 2 h in 1 mL of PBS containing 1% bovine serum albumin (Research Products International, A30075) and 0.5 mM PMSF (Research Products International, P20270) (PBS/BSA). Unbound antibody was removed by successive washes with PBS/BSA, and the antibody bound Protein A/G PLUS agarose was added to 900 μL of culture medium lysate or 200 μg of cellular lysate. Following overnight incubation at 4°C, the immunoprecipitated CA-antibody complexes were pelleted at 5,000xg for 4 minutes and washed three times in IP wash buffer (10 mM Tris pH 7.4, 150 mM NaCl, 1% Triton X-100, 1 mM EDTA, 0.5 mM PMSF). CA immunoprecipitates were eluted into 40 μL of 2x sample loading buffer (62.5 mM Tris pH 6.8, 15% glycerol, 2% SDS, 0.004% bromophenol blue, and 100 mM 2-mercaptoethanol) by heating to 95°C for 5 minutes.

### Cell lysis, immunoblotting, and phosphorimaging

Cells lysates were lysed and protein concentration quantified for immunoblotting as described by reference [47]. An equivalent to 20 ng of p24 (Fig 5C and 5E), 70 ng of p24 (Fig 5G) or 15 μg of total protein were loaded per well onto a 4 to 20% Bis-Tris SDS PAGE gel (Genscript). For immunoblotting of ^35^S-labeled CA immunoprecipitants of cellular lysates, 15 μL of immunoprecipitate elution was loaded on 4 to 20% Bis-Tris SDS PAGE gel (Genscript). For CA immunoprecipitated from released HIV-1 particles, the cellular lysate protein concentration was used to calculate the volume loaded in each well. Gels were transferred onto PROTRAN nitrocellulose membranes (Perkin Elmer) using a Trans-Blot SD semi-dry transfer cell (BioRad). Membranes were probed using anti-CA (183-H12-5C, NIH AIDS Repository, 1513, 0.4 ng/ μL), human HIV-Ig (NIH AIDS Repository, 3957, 1:10,000), anti-MA (NIH AIDS Repository, 4811, 1:500), rabbit anti-Flag (Cell Signaling, 14793, 1:1000), rabbit anti-IN (Rockland Immunochemicals, Inc, 103797, 1:2000), or mouse anti-β-actin (Sigma, A2228, 1:50,000). The appropriate secondary fluorescent conjugated antibodies were incubated with the blot, and the membrane was imaged using a LI-COR Odyssey. Li-COR Image Studio 3.1 was used to quantify the fluorescence intensity of each band in Fig 5C, 5E and 5G. In Fig 5G, the percent normalized signal was calculated by first dividing the IN or RT p66 signal by the respective CA-specific signal, the signal obtained from probing the blot with a primary antibody against CA. The resulting values were then divided by the average IN signal/ CA signal of particles released from WT MT-4 cells.

For ^35^S detection, each immunoblot was exposed to a storage phosphor screen (Amersham Biosciences), and the stored signal was detected using a Typhoon Trio variable-mode imager (GE Biosciences). Signal intensity for each band was quantified using ImageQuant 5.2 (Molecular Dynamics). Background signal for each lane was subtracted the respective band prior to calculating the fraction of signal for each Gag processing intermediate or the total CA signal per lane. Correction for differences in transcription/ translation efficiencies between corresponding complemented and KO_Vector_ cell lines was as follows: For each chase time point in S6B and S6C Fig, the total CA signal for the KO_Vector_ cell lines was divided by the total CA signal for the respective complemented cell line to yield the Gag transcription/ translation efficiency. For each timepoint, the signals of the particles released from KO_Vector_ cell lines were adjusted by dividing the total CA signal from released virions by the respective Gag transcription/ translation efficiency. The C5 IPMK KO_Vector_ cell line was 26.5 +/- 1.9% as efficient at Gag transcription/translation compared to the C5 IPMK KO_IPMK-Flag_ line. IPPK KO had little effect on Gag transcription/ translation efficiency, and the KO_vector_ line was 89 +/- 0.5% as efficient as the B6 IPPK KO_Flag-IPPK_ line.

### Statistics

Excluding box plots, all error bars represent standard deviation of the mean. Prior to testing to compare between two groups, each group of samples was compared using a one-way ANOVA using the data analysis tools add in within Excel. If p<0.05 for the ANOVA, samples were compared to WT using a one- or two-way Dunnett’s test using the multcomp package in R. For Figs 1H, 2D, 2E, 6D–6O and S8, the data for the complemented clones is compared to the respective KO_Vector_ clone using Welch’s t-Test in R. In Fig 5H, Fisher’s Exact Test was used to compare virion morphologies between WT and IPMK KO cell lines.

## Supporting information

S1 Fig IPPK alleles. A–C. Alignment of the indicated *IPPK* alleles from WT and *IPPK* KO cells. Alleles A, B, and C of CEM G4 IPPK KO cells refer to the three, distinct fusion alleles present in the clone.
(TIF)

S2 Fig IPMK alleles. A–C. The denoted *IPMK* alleles are aligned for WT and *IPMK* KO cells. For MT-4 C6 IPMK KO cells, three alleles were identified by sequencing and agarose gel electrophoresis denoted as allele A, B, and C.
(TIF)

S3 Fig HIV-1 viral spread in MT-4 KO panel. A. and D. p24 release from cells infected HIV-1_NL4-3_. In (A) and (D), 100,000 or 1,000,000 cells were infected with 1 or 180 ng p24 of HIV-1_NL4-3_, respectively. B and G. Histograms of Gag fluorescence intensity for the specified dpi and cell types. C and F. Plot of the average Gag MFI. E. The fraction of infected cells over time in the indicated cell lines by immunostaining for Gag. H. Box blot of CD4 expression by flow cytometry for each cell line. Box shows the 25^th^ to 75^th^ quartiles with the line denoting the median. Outlier points are shown as dots. The same HIV-1 MOI is used in panels A–C, 1 ng p24 per 100,000 cells. In panels D–G, the cells were infected with 180 ng p24 HIV-1 per 1,000,000 cells. HIV-1 release in panels (A) and (D) are from 4 independent infections. Each bar in panels (C), (E), and (F) represents the average of 3 independent experiments. Significance levels: * p<0.05, ** p<0.01, *** p<0.001, **** p<0.0001.
(TIF)

S4 Fig HIV-1 viral spread in CEM IPMK or IPPK KO clones. A and B. Top panel: Diagram of *IPPK* or *IPMK*, respectively. Red arrows denote approximate Cas9 cleavage site. Genomic PCR primer locations are indicated in black and orange. Primer locations are not exact. *IPMK* exon 1 expected PCR product size: 278 bp. *IPPK* exon 1 expected PCR product size: 355 bp. *IPPK* exon 1 to 7 fusion allele expected PCR product size: 397 bp. *IPMK* exon 1 to 6 fusion allele expected PCR product size: 252 bp. Bottom panels: Amplified genomic DNA from the designated cell lines run on an ethidium bromide stained agarose gel. C and D. Toluidine blue stained polyacrylamide gel of TiO_2_ enriched inositol phosphates. E and F. HIV-1 p24 release graphed versus time for the specified HIV-1_NL4-3_-infected CEM cell lines. Each point represents the average of 4 independent infections. Asterisks signify significance from WT CEM cells. G and H. CD4 expression by flow cytometry in the indicated WT and KO CEM clones graphed on a box and whiskers. Box shows the 25^th^ to 75^th^ quartiles with the line denoting the median. Dots denote outliers. Significance levels: * p<0.05, ** p<0.01, *** p<0.001, **** p<0.0001.
(TIF)

S5 Fig Intracellular Gag accumulation in IPMK or IPPK KO cells. A, C, E. Normalized Gag MFI from flow cytometry for the denoted cell lines. Each bar represents 3 independent experiments. Asterisks signify significance from WT cells. B, D, F. Box and whiskers plots of Gag Fluorescence intensity by flow cytometry for the high MOI inocula shown in (A), (C), or (E), respectively. Box shows the 25^th^ to 75^th^ quartiles with the line denoting the median. Significance levels: * p<0.05, ** p<0.01, *** p<0.001.
(TIF)

S6 Fig Electron microscopy of HIV-1 produced by IPMK or IPPK KO MT-4 cells. A–E. Representative micrographs of (A) WT, uninfected MT-4 cells, (B) HIV-1_NL4-3_ infected WT MT-4 cells, (C) HIV-1_NL4-3_ infected B6 IPPK KO cells, (D) HIV-1_NL4-3_ infected C5 IPMK KO cells, or (E) HIV-1_NL4-3_ infected C6 IPMK KO cells. The left panel shows a 11,000 X magnification image and the right panel depicts a high magnification micrograph of the boxed area. Cyan arrowhead: immature virion. Green arrowhead: aberrant virion. Red arrowhead: mature virion, top view. Orange arrowhead: mature virion, side view. Purple arrowhead: eccentric virion. Box boundaries shown in the left panel are not exact.
(TIF)

S7 Fig CA immunoprecipitation from ^35^S-labeled HIV-1-infected cell lysates. A–C. Immunoblots of CA immunoprecipitants from the indicated HIV-1-infected MT-4 cell lines pulse labeled with ^35^S and chased for the indicated time in hours (hr). Top panel: Image of ^35^S labeling. Bottom panel: Image of blot probed with HIV-Ig. Markers on the left denote protein sizes in kiloDaltons. D–I. Quantification of ^35^S labeled intracellular Gag cleavage during the chase period. Top graph shows first independent experiment. Bottom panel contains the second independent experiment. Cellular lysates were used in the CA immunoprecipitation in panels A–I. X-axis shows the time of chase in hours.
(TIF)

S8 Fig Effect of back complementation of IPPK or IPMK on HIV-1 release. A. and B. Graphs of ^35^S labeled HIV-1 release from the indicated cell lines. Points represent the average of 2 independent experiments. Points are not corrected for differences in Gag transcription/ translation efficiency between corresponding KO_Vector_ and back complemented cell lines. Significance levels: * p<0.05, ** p<0.01.
(TIF)

S1 Table Genomic PCR of IPMK or IPPK locus. (XLSX)

S1 File Supporting data. (ZIP)
