# Correction: Inositol phosphates promote HIV-1 assembly and maturation to facilitate viral spread in human CD4^+^ T cells

**DOI:** 10.1371/journal.ppat.1009389

**Published:** 2021-03-02

**Authors:** Gregory A. Sowd, Christopher Aiken

The following information is missing from the Funding statement: This study was supported by NIH grant P50 AI150481.

The full funding statement should read:

This study was funded by National Institutes of Health (https://www.nih.gov/) grants R21 AI150384, R56 AI076121, and P50 AI150481 to CA. The funders had no role in study design, data collection and analysis, decision to publish, or preparation of the manuscript.
