# Supplementary figures and images for: Inositol phosphates promote HIV-1 assembly and maturation to facilitate viral spread in human CD4+ T cells
Source: PLoS Pathog. 2021 Jan 21;17(1):e1009190. doi: 10.1371/journal.ppat.1009190 (PMC7853515; doi:10.1371/journal.ppat.1009190)

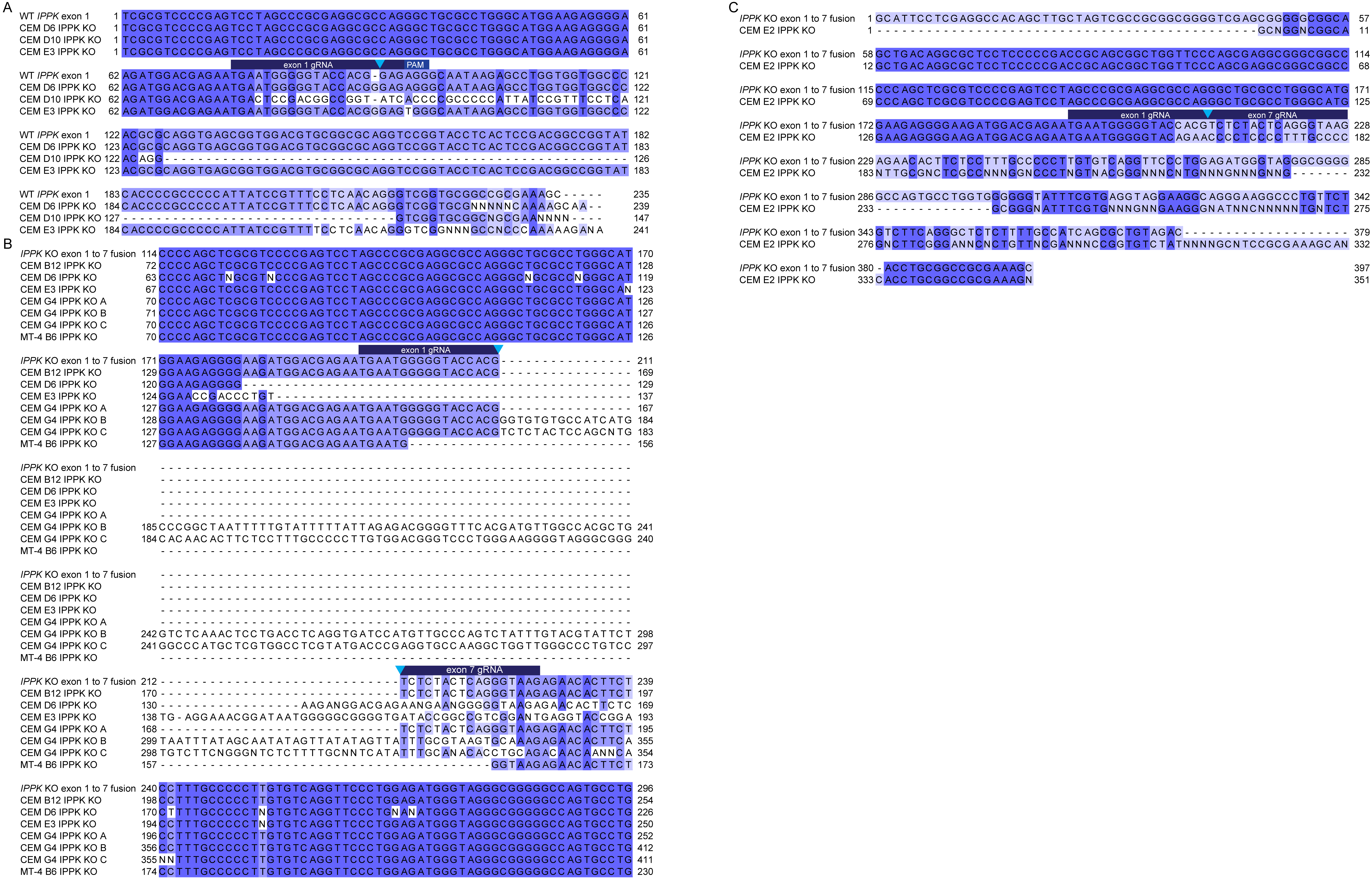

Supplement: S1 Fig — A–C. Alignment of the indicated IPPK alleles from WT and IPPK KO cells. Alleles A, B, and C of CEM G4 IPPK KO cells refer to the three, distinct fusion alleles present in the clone. (TIF) [file ppat.1009190.s001.tif]

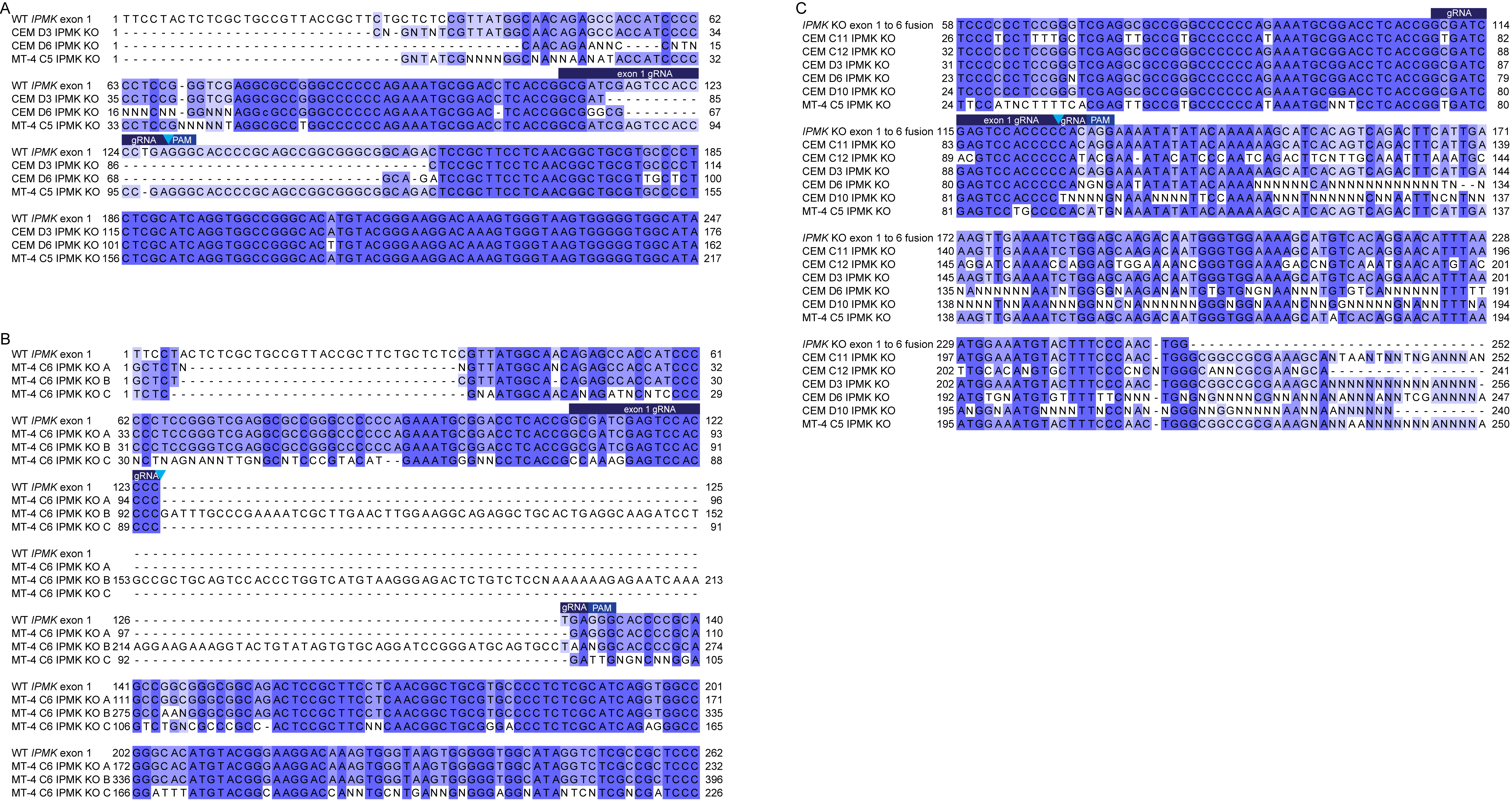

Supplement: S2 Fig — A–C. The denoted IPMK alleles are aligned for WT and IPMK KO cells. For MT-4 C6 IPMK KO cells, three alleles were identified by sequencing and agarose gel electrophoresis denoted as allele A, B, and C. (TIF) [file ppat.1009190.s002.tif]

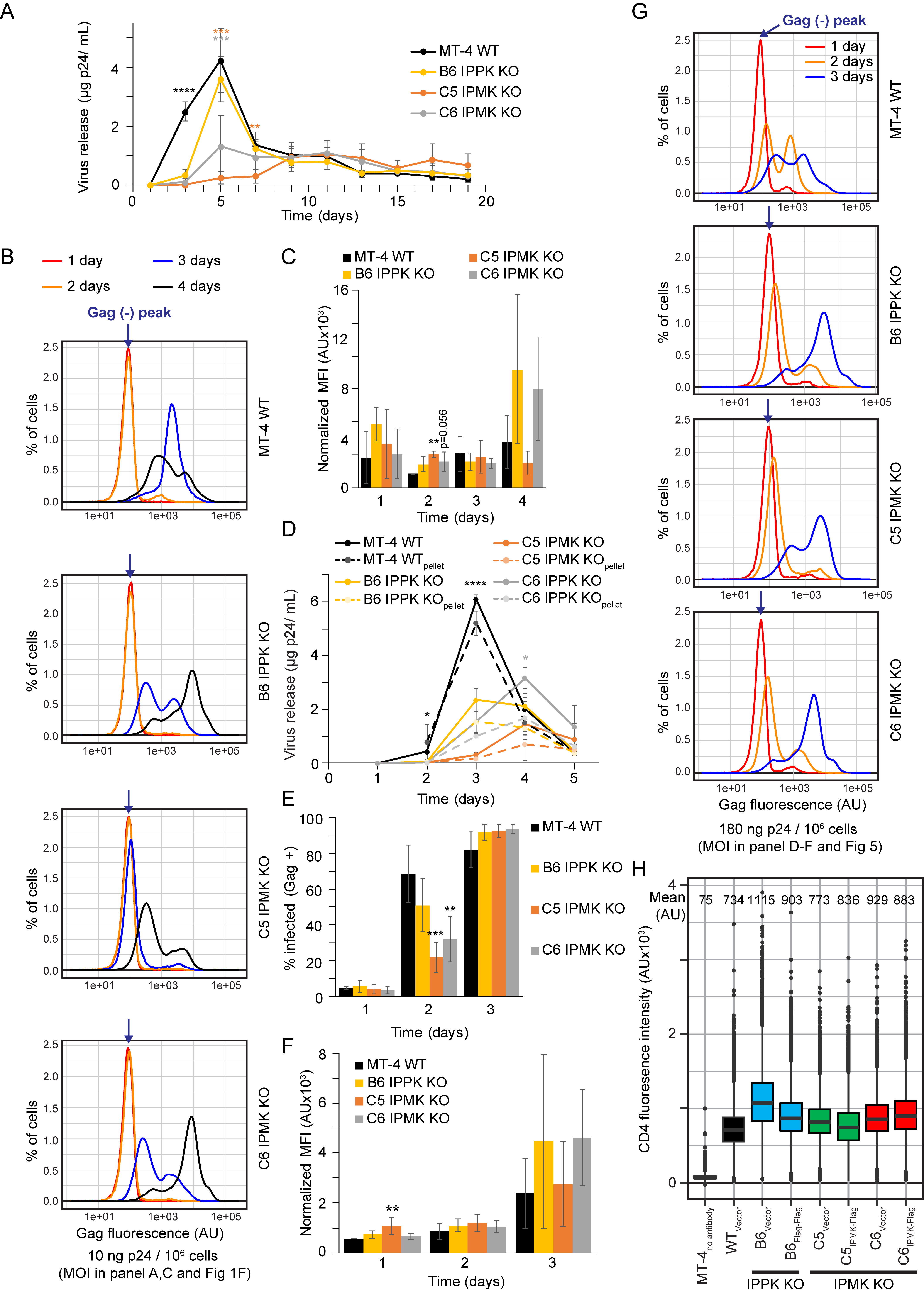

Supplement: S3 Fig — A. and D. p24 release from cells infected HIV-1NL4-3. In (A) and (D), 100,000 or 1,000,000 cells were infected with 1 or 180 ng p24 of HIV-1NL4-3, respectively. B and G. Histograms of Gag fluorescence intensity for the specified dpi and cell types. C and F. Plot of the average Gag MFI. E. The fraction of infected cells over time in the indicated cell lines by immunostaining for Gag. H. Box blot of CD4 expression by flow cytometry for each cell line. Box shows the 25th to 75th quartiles with the line denoting the median. Outlier points are shown as dots. The same HIV-1 MOI is used in panels A–C, 1 ng p24 per 100,000 cells. In panels D–G, the cells were infected with 180 ng p24 HIV-1 per 1,000,000 cells. HIV-1 release in panels (A) and (D) are from 4 independent infections. Each bar in panels (C), (E), and (F) represents the average of 3 independent experiments. Significance levels: * p<0.05, ** p<0.01, *** p<0.001, **** p<0.0001. (TIF) [file ppat.1009190.s003.tif]

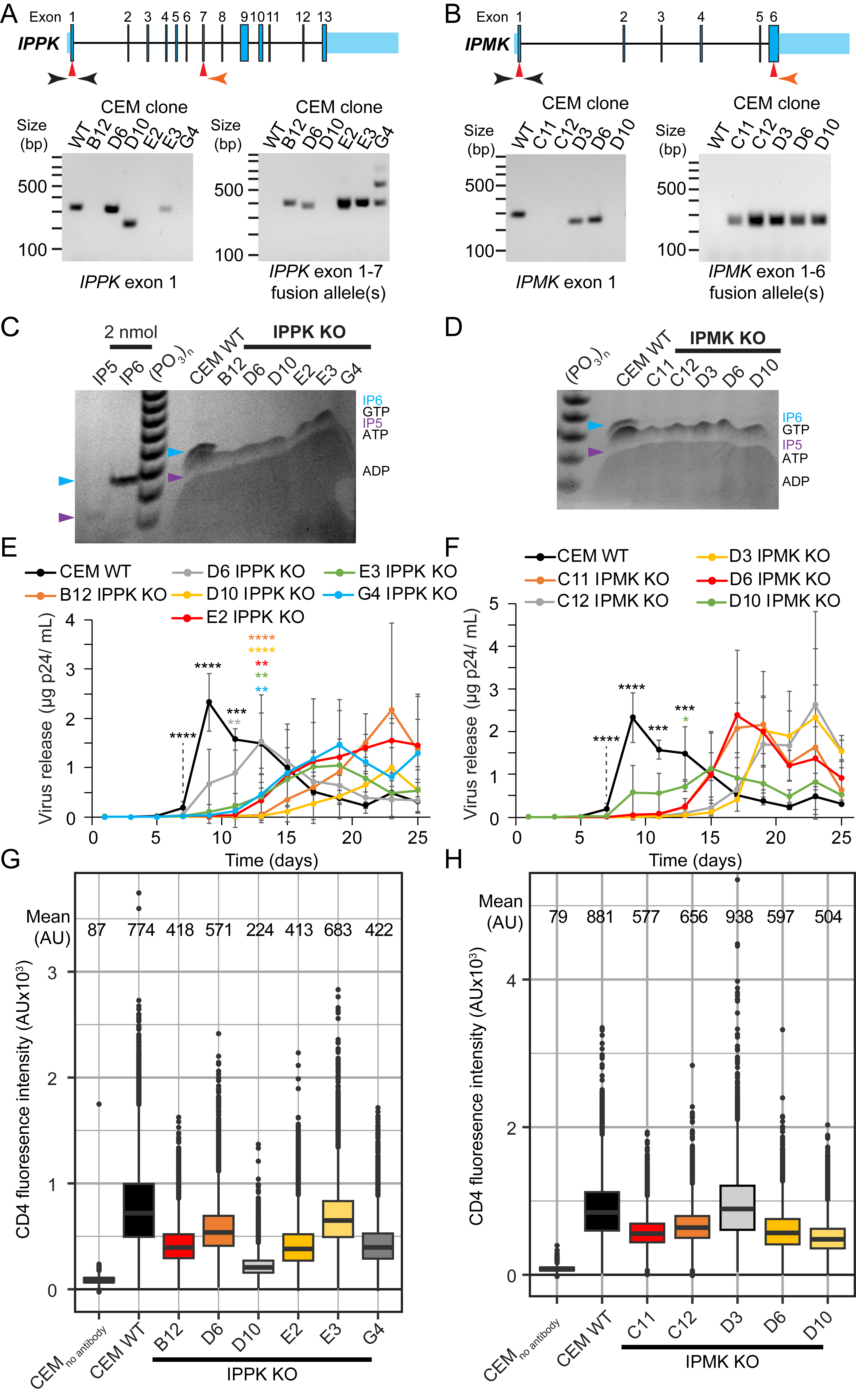

Supplement: S4 Fig — A and B. Top panel: Diagram of IPPK or IPMK, respectively. Red arrows denote approximate Cas9 cleavage site. Genomic PCR primer locations are indicated in black and orange. Primer locations are not exact. IPMK exon 1 expected PCR product size: 278 bp. IPPK exon 1 expected PCR product size: 355 bp. IPPK exon 1 to 7 fusion allele expected PCR product size: 397 bp. IPMK exon 1 to 6 fusion allele expected PCR product size: 252 bp. Bottom panels: Amplified genomic DNA from the designated cell lines run on an ethidium bromide stained agarose gel. C and D. Toluidine blue stained polyacrylamide gel of TiO2 enriched inositol phosphates. E and F. HIV-1 p24 release graphed versus time for the specified HIV-1NL4-3-infected CEM cell lines. Each point represents the average of 4 independent infections. Asterisks signify significance from WT CEM cells. G and H. CD4 expression by flow cytometry in the indicated WT and KO CEM clones graphed on a box and whiskers. Box shows the 25th to 75th quartiles with the line denoting the median. Dots denote outliers. Significance levels: * p<0.05, ** p<0.01, *** p<0.001, **** p<0.0001. (TIF) [file ppat.1009190.s004.tif]

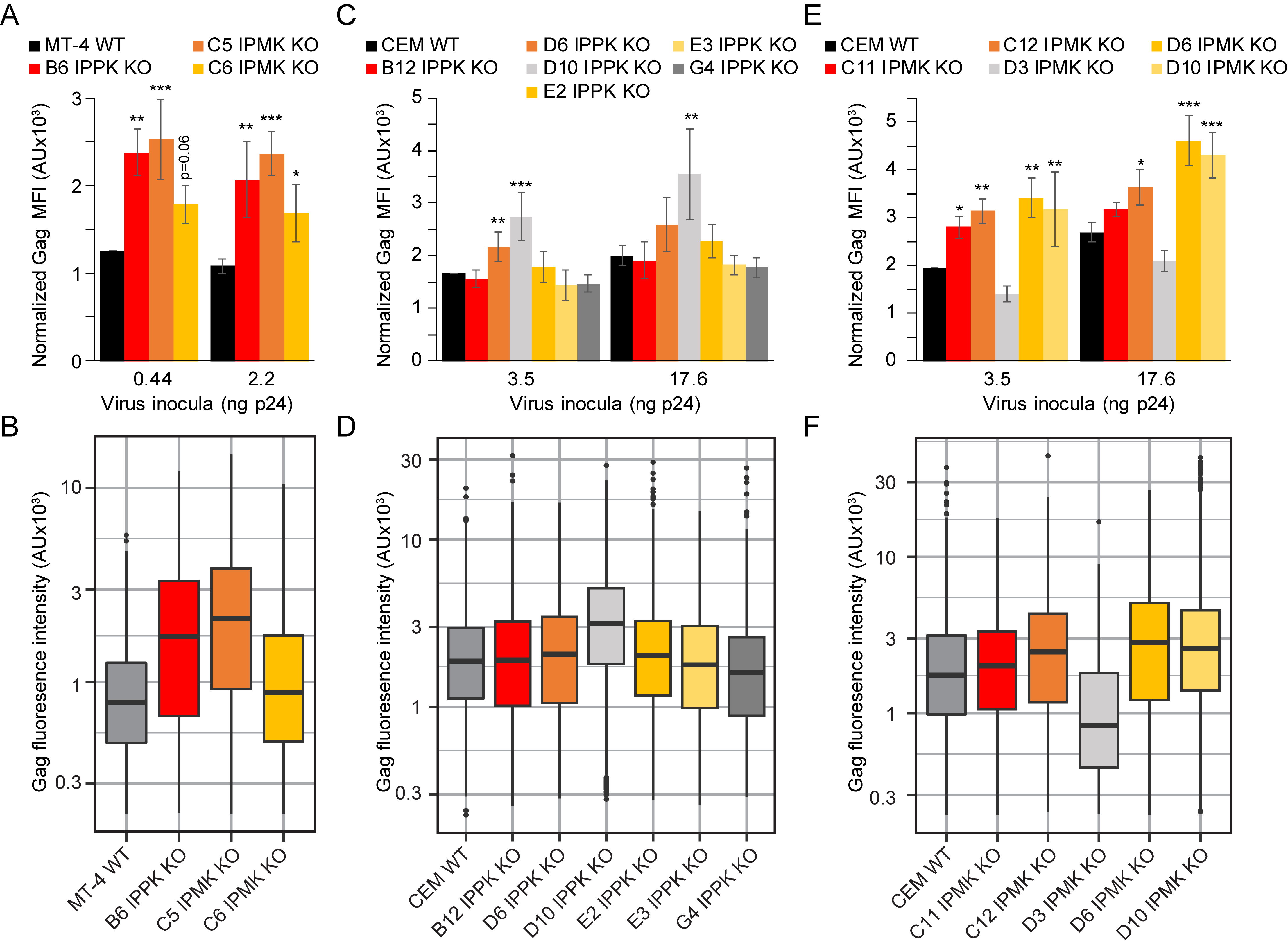

Supplement: S5 Fig — A, C, E. Normalized Gag MFI from flow cytometry for the denoted cell lines. Each bar represents 3 independent experiments. Asterisks signify significance from WT cells. B, D, F. Box and whiskers plots of Gag Fluorescence intensity by flow cytometry for the high MOI inocula shown in (A), (C), or (E), respectively. Box shows the 25th to 75th quartiles with the line denoting the median. Significance levels: * p<0.05, ** p<0.01, *** p<0.001. (TIF) [file ppat.1009190.s005.tif]

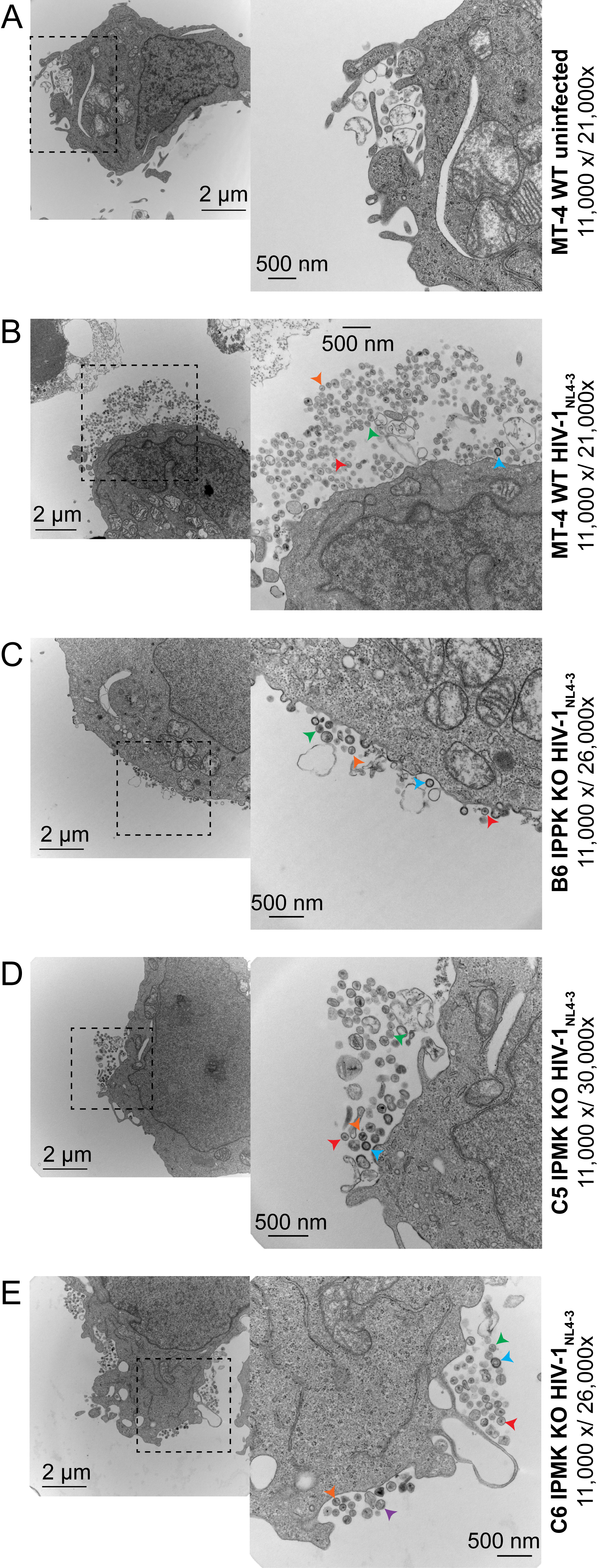

Supplement: S6 Fig — A–E. Representative micrographs of (A) WT, uninfected MT-4 cells, (B) HIV-1NL4-3 infected WT MT-4 cells, (C) HIV-1NL4-3 infected B6 IPPK KO cells, (D) HIV-1NL4-3 infected C5 IPMK KO cells, or (E) HIV-1NL4-3 infected C6 IPMK KO cells. The left panel shows a 11,000 X magnification image and the right panel depicts a high magnification micrograph of the boxed area. Cyan arrowhead: immature virion. Green arrowhead: aberrant virion. Red arrowhead: mature virion, top view. Orange arrowhead: mature virion, side view. Purple arrowhead: eccentric virion. Box boundaries shown in the left panel are not exact. (TIF) [file ppat.1009190.s006.tif]

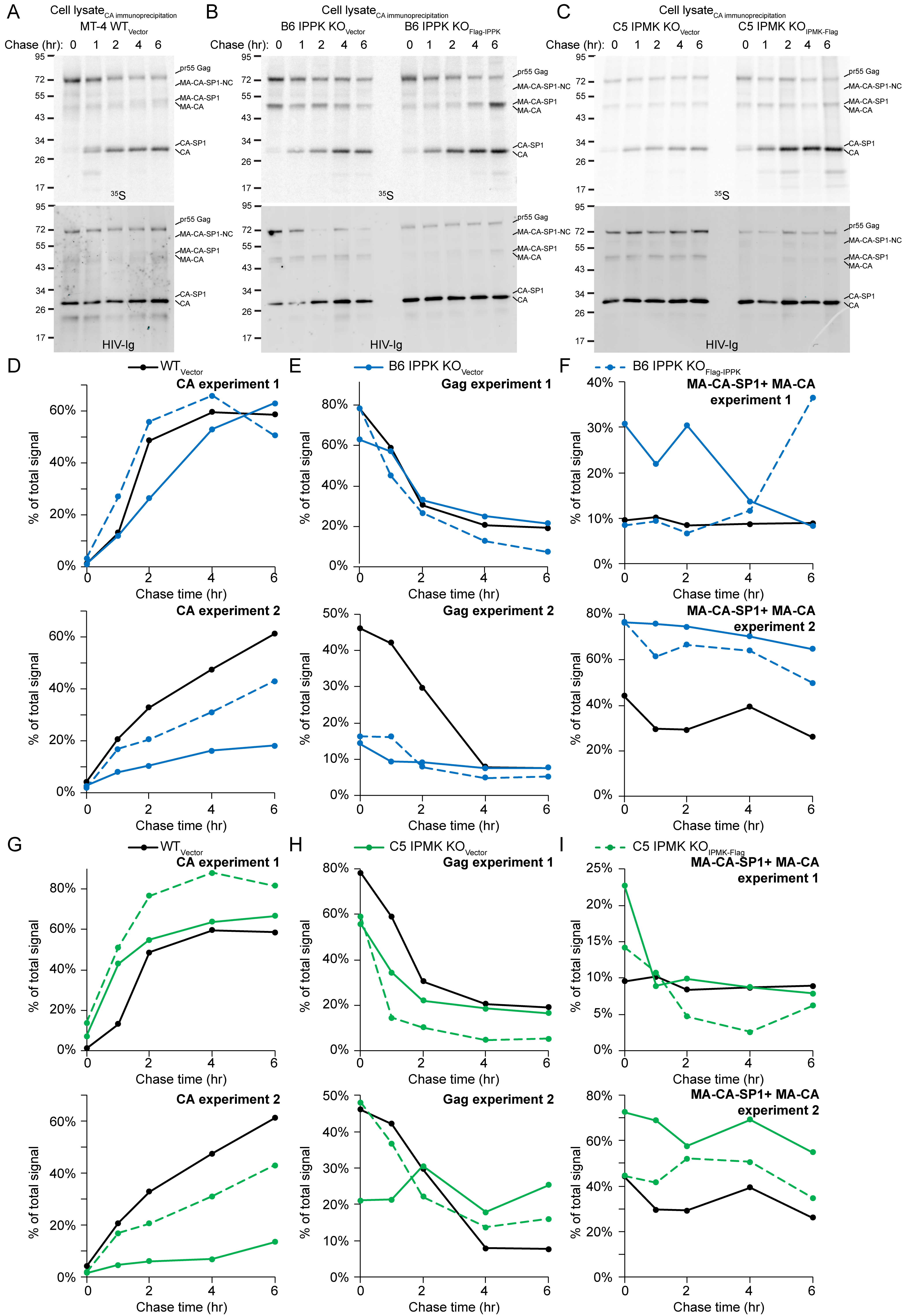

Supplement: S7 Fig — A–C. Immunoblots of CA immunoprecipitants from the indicated HIV-1-infected MT-4 cell lines pulse labeled with 35S and chased for the indicated time in hours (hr). Top panel: Image of 35S labeling. Bottom panel: Image of blot probed with HIV-Ig. Markers on the left denote protein sizes in kiloDaltons. D–I. Quantification of 35S labeled intracellular Gag cleavage during the chase period. Top graph shows first independent experiment. Bottom panel contains the second independent experiment. Cellular lysates were used in the CA immunoprecipitation in panels A–I. X-axis shows the time of chase in hours. (TIF) [file ppat.1009190.s007.tif]

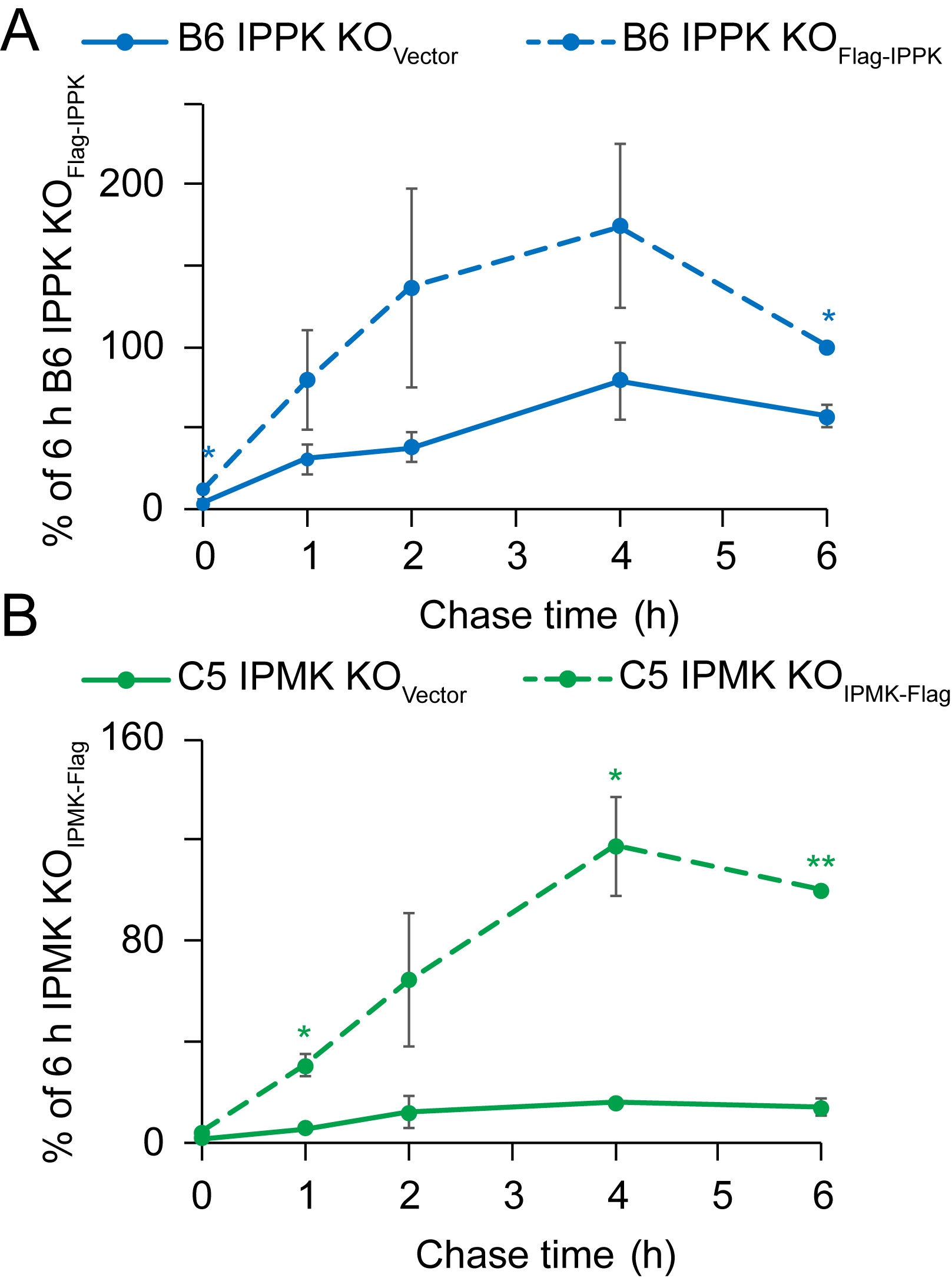

Supplement: S8 Fig — A. and B. Graphs of 35S labeled HIV-1 release from the indicated cell lines. Points represent the average of 2 independent experiments. Points are not corrected for differences in Gag transcription/ translation efficiency between corresponding KOVector and back complemented cell lines. Significance levels: * p<0.05, ** p<0.01. (TIF) [file ppat.1009190.s008.tif]
